# Priorities for change for autistic people across Europe

**DOI:** 10.1186/s13229-026-00706-3

**Published:** 2026-02-19

**Authors:** Siti Nurnadhirah Binte Mohd Ikhsan, Rosemary Holt, Amber Ruigrok, Joyce Man, Tracey Parsons, Kathryn Gibbs, Edward Bullock, Aurélie Baranger, Carrie Allison, Mary Doherty, Anjuli Ghosh, Jerneja Terčon, Katrien Van den Bosch, Simon Baron-Cohen

**Affiliations:** 1 Autism Research Centre, Department of Psychiatry, University of Cambridge, Douglas House, 18b Trumpington Road, Cambridge, CB2 8AH UK; 2 Division of Psychology and Mental Health, School of Health Sciences, Faculty of Biology, Medicine and Health, University of Manchester, Manchester, UK; 3 Autism-Europe, Brussels, Belgium; 4 A-Reps AIMS-2-TRIALS Consortium, Brussels, Belgium; 5 University College Dublin, Dublin, Ireland

## Abstract

**Background:**

Despite rising rates of autism prevalence, there remains a pressing need to enhance the quality of life for autistic people in Europe and around the world.

**Methods:**

We conducted the *10 Points for Change* survey to identify the 10 most important areas that require improvement for autistic people across the region. Data from 1,709 autistic people, parents/carers and members of autism-related organisations residing within the European Union (EU) and the United Kingdom (UK) were analysed, together with autism-related differences (autistic vs. non-autistic; formal vs. no formal autism diagnosis) and gender differences (male vs. female) in results.

**Results:**

Across groups, areas that require the most urgent changes are education, public awareness and understanding of autism, employment, and government funding for autism-specific services. Differences in results between groups reflect their specific needs and experiences. Discrimination is a crucial area for change according to autistic people with formal diagnosis of autism, whereas autistic people without formal diagnosis indicate diagnostic services as a priority for change. According to parents/carers and members of autism-related organisations, changes are also needed to improve social inclusion of autistic people. Other areas of priority for change across all groups include mental healthcare (within top 10 for autistic participants and parents/carers), support with daily living, and post-diagnostic services (the latter two within top 10 for parents/carers and members of autism-related organisations). For some areas, their identification and importance as priorities for change significantly varied with whether participants were autistic or formally diagnosed and autistic participants’ gender. Comparisons across countries with the greatest representation in the survey – Germany, the UK, France, Spain and Poland – revealed consistent priorities.

**Limitations:**

Consideration should be given to issues related to methodology and data availability such as how representative the sample is of countries across the EU and the UK. Autistic people with high support needs might have also been unable to participate directly and responses from carers representing them might not fully reflect their views or provide representative data.

**Conclusion:**

Change through concerted legislative actions within and across countries in Europe is needed to address the priority areas for change for autistic people.

**Supplementary Information:**

The online version contains supplementary material available at 10.1186/s13229-026-00706-3.

## Background

Autistic people in Europe and other parts of the world continue to face challenges in various aspects of life [1]. From education [2] and employment [3], to support with daily living [4] and social inclusion [5], autistic people are still not able to access rights and services that meet their needs [6]. In cases where services can be accessed, quality and conditions are often still inadequate for autistic people [7, 8] and may even be detrimental to their wellbeing [9]. Family members and carers of autistic people also report problems acquiring the services needed by their autistic relative [6] as well as their own personal struggles as caregivers [10]. In addition, professionals working in the field of autism describe limitations that prevent them from being able to serve autistic people well such as lack of resources [11] and adequately trained staff [12].

Addressing the needs of autistic people is crucial given their complex physical [13] and psychological [14] needs. Autistic people are more likely than non-autistic people to have had negative life experiences such as challenges with education, unemployment, domestic abuse, victimisation, financial exploitation and hardship [15, 16]. In addition, autistic people are disproportionately subjected to bullying, abuse and discrimination [17]. These adverse experiences are associated with higher rates of anxiety and depression, which are more prevalent among autistic people than non-autistic people [14, 18]. Whilst autistic people and carers generally prefer using services that cater specifically to autistic people (e.g. early intervention; on-the-job training for autistic staff) to more generic services [19], and autism-specific services are usually more sensitive and effective in addressing autistic people’s needs, both types of support should be offered to the autistic community. This is especially relevant since certain services that cater to the wider public are typically more accessible, affordable and available. Autistic students, for example, may be directed to general tutoring assistance if it aligns with their needs, considering that this service is often more available and subsidised than specialised tutoring tailored for autistic learners. There should also be greater effort in making autism-specific services more mainstream in order to improve their accessibility and affordability, promote inclusivity of autistic people, reduce stigma and offer support in more areas, though specialised support remains necessary for some autistic people.

Policies and strategies, both within countries and across Europe, are required for implementing the changes necessary to improve autistic people’s wellbeing and experiences. In support of this, the European Parliament adopted a resolution that called on the European Commission and European Union (EU) Member States to protect the rights of autistic people in Europe [20]. For example, in Spain, the Spanish Strategy for Autism Spectrum Disorders [21] has outlined actions at the state and national level that aim to enhance autistic people’s quality of life and protect their rights. Other examples include the UK, which have developed a national strategy for autistic children, young people and adults with the objective of improving the lives of autistic people, carers and families [22], and Malta, where an autism advisory council chaired by an autistic lawyer has drafted the 2021–2030 National Autism Strategy as part of the 2016 Autism Spectrum (Empowerment) Act [23, 24]. However, despite efforts by governments and lawmakers at all levels, inefficiencies in policy implementation remain a concern and autistic people still face challenges in their everyday life. For instance, the progress of the Spanish strategy was stalled several times since it was enacted in 2015 due to successive changes in government [25], and while Spanish law mandates that companies with more than 50 employees hire at least 2% of staff with disabilities, not all businesses conform to it and prefer to be fined rather than take in disabled workers [26]. Nevertheless, legislative action is still a valuable means of safeguarding the rights of autistic people and their families as well as improving their experiences.

We have therefore conducted the *10 Points for Change* study to examine areas that are important to the autism community in Europe, taking into account key factors such as gender and autism diagnosis. Understanding the priorities for change helps society and policymakers understand where change is most needed and can help inform the design and implementation strategy leading to more effective autism policies. An online survey was distributed to autistic people, family carers and autism service professionals living within the EU and the UK and explored participants’ priorities for change for autistic people and their ranking of these priorities. Differences in priorities across countries and current autism policies in the region were also analysed.

As it is imperative to develop a common strategy across Europe on autism, and given that consultation with members of the autistic community is vital in informing autism policies, we sought to convey findings from the *10 Points for Change* survey to EU policymakers. The top 10 priorities for change for the autism community in Europe were used to develop communication materials which were then presented to policymakers to raise awareness of key areas where policy change could bring about a positive impact on the lives of autistic people and their families.

## Methods and materials

### Survey development

To shortlist the top priorities for change to include in the survey, two focus groups were conducted with representatives of the autism community. One focus group included five autistic people and the other was comprised of four parents of autistic people. Members of these groups are of diverse backgrounds and age groups, often interacted personally or worked professionally with other autistic people, and resided in eight countries across Europe. They were recruited from the Autism Innovative Medicine Studies-2-Trials (AIMS-2-TRIALS) Autism Representatives (A-Reps) group, which consisted of autistic people and parents of autistic people living in the EU and the UK. This group was set up at the start of the AIM-2-TRIALS consortium to ensure community input and has worked with the consortium researchers throughout the project. A-Reps were also invited to be co-authors on the paper.

During each focus group discussion, autistic participants were asked which areas in their lives required improvement and concerned them as autistic people, while family members and carers of autistic people were asked the same in relation to their autistic relative. All participants were also asked about policies that they feel should be adopted by their country to improve these areas. We also reviewed studies with a similar research focus as the *10 Points for Change* study and explored the goals of autism associations in Europe such as Autism-Europe (AE), an umbrella association of autism organisations across the region, as they reflect what the associations believe are crucial for them to advocate and change for autistic people. In addition to reviewing existing literature and autism support organisation websites, thematic analysis using NVivo [27] was conducted to establish themes from the focus group transcripts [28]. Each phase of the analysis was reviewed by two members of the research team, and any disagreements were resolved through discussion until agreement was reached. One of the researchers identified as autistic, the other was a clinical psychologist, and during this analysis, both resided in one of the target countries of the study i.e. the UK. Nineteen themes, or areas of priority for change, were shortlisted as a result (see **Methods and Materials – Measures**) and collated into a survey to establish autistic people, parents/carers, and members of autism-related organisations’ priorities for change. A follow-up meeting was held with members of the focus groups to review and check the accuracy of themes that came out of initial theme-setting focus groups. This feedback meeting also included further discussion on the design, optimisation and the accessibility of the survey for autistic people.

The survey was conceived in English using Qualtrics and translated, along with recruitment materials (see Supplementary Fig. 1), into Czech, French, German, Italian, Polish, Slovenian and Spanish by a certified translation agency. French, German, Italian and Spanish were chosen as they are some of the most commonly spoken languages in Europe [29]. Czech, Polish and Slovenian were indicated by most participants in a previous survey of autistic people in Europe, the ACCESS-EU study [30], as languages they would like the survey to be available in. Native speakers among A-Reps and research partners such as AE were involved in checking the accuracy of the translations and whether they were culturally sensitive when describing autism.

The survey is available to view on the Open Science Framework (OSF) at https://osf.io/ucfhz/overview?view_only=c5d4b7d519f24e028d5b74f05289ab3f.

### Participants

Priorities survey participants were autistic people, parents, carers and members of autism-related organisations such as charities and disability and research organisations. However, people working in the wider field of autism (including volunteers) might also identify as members of autism-related organisations and participate in the survey, as specified by the recruitment poster (see Supplementary Fig. 1). Participants should also be residents of EU Member States or the UK and aged 16 years old or above.

Autistic people who were unable to complete the survey on their own, due to their young age or relatively high support needs, were also represented in the study. Parents or carers were allowed to assist in inputting their autistic family members’ responses in the survey. Survey entries completed by their parents or carers were expected to reflect the views and experiences of their autistic family members.

All respondents were allowed to withdraw from the survey at any time. The *10 Points for Change* study was approved by the Cambridge Psychology Research Ethics Committee (reference number PRE.2020.071).

### Measures

All participants filled in the same version of the survey.

The survey questions are outlined in Supplementary Fig. 2. Following consent, participants were asked to indicate if they completed the survey on their own or with support. To obtain information on participant demographics, participants were then asked to indicate their age, gender (‘male’, ‘female’, ‘non-binary’ or ‘other’, with only one selection allowed), country of residence, and whether they were: (1) autistic – formally diagnosed by a professional, (2) autistic – self-diagnosed, (3) autistic – awaiting assessment, (4) parent or family carer of an autistic person, or (5) member of an autism-related organisation. Participants could indicate multiple options.

Participants were then asked to (1) indicate any of 19 areas (see Supplementary Table 1) that, according to their experiences, needed to change for autistic people, (2) briefly explain their experiences in the selected areas, (3) describe any suggestions for improvements in the selected areas, and (4) rank the areas in descending order of importance. The first section was a multiple-choice question while questions 2 and 3 were open-ended, allowing free text responses. This paper focuses solely on quantitative data from questions 1 and 4. For question 4, participants were shown the list of areas and asked to drag and drop them in order of importance such that the most important areas were at the top and the least important ones were at the bottom. The order of the list was consistent across all participants. Descriptors for the areas of priority for change (see Supplementary Table 1) were shown to participants during the survey. Descriptors for the areas of priority for change (see Supplementary Table 1) were shown to participants during the survey. All areas had to be ranked by all participants except ‘Other’ area; only participants who had selected it as a priority for change in question 1 were asked to rank it.

If ‘Other’ was selected, participants were asked to briefly describe what this area was and rank this area.

### Survey distribution

The survey was first launched in November 2020 and remained open until February 2024. It was disseminated through various local and international avenues including the Cambridge Autism Research Database (CARD), the AIMS-2-TRIALS and Autism Research Centre official websites and Twitter/X accounts. The survey was circulated by autism research institutes in European universities and hospitals within the AIMS-2-TRIALS consortium, as well as autism organisations and charities across the EU and the UK. AIMS-2-TRIALS partner institutions were asked to help distribute the survey to their networks within their countries. AE advertised the survey in their newsletters and social media platforms to their members across multiple countries. A-Reps also distributed the survey to their networks within their countries of residence. Recruitment was more targeted towards countries where languages which the survey was available in were widely spoken – English, Czech, French, German, Italian, Polish, Slovenian and Spanish – and where our recruitment sites as well as partners were located. Furthermore, in the latter stages of recruitment, efforts were focused on countries with lower numbers of participants.

### Data analysis

Data from survey entries that at least completed question 1, which was on areas that needed to change for autistic people, were analysed. Survey entries that lasted less than 120s were excluded from analysis to remove possible bots. Non-English free text survey entries were translated into English using Google Translate [31], and if the resulting translations were unclear, Microsoft Translator [32] and DeepL Translator [33] were used.

Only quantitative data obtained by the survey are presented in this paper, namely participants’ demographic information, responses as to which of the areas were important for change, and their rankings of these areas in order of importance for change. Qualitative data are being analysed at the time of publication with the intention to publish these separately.

Because multiple selections were allowed in response to the question of whether participants were (1) formally diagnosed with autism by a professional, (2) self-diagnosed, (3) awaiting assessment, (4) parent or family carer of an autistic person, or (5) member of an autism-related organisation, participant responses had to be re-evaluated to categorise participants into more definitive groups. This particularly concerns responses about autism diagnosis, where participants might provide contradictory answers (e.g. selecting both ‘formal diagnosis’ and ‘self-diagnosed’ options). Hence, we defined participants with a formal diagnosis of autism as all respondents indicating as such, regardless of whether they had said that they were also self-diagnosed or awaiting assessment, while autistic respondents with no formal diagnosis were ascertained as those who were self-diagnosed, awaiting assessment, or both, and were not formally diagnosed.

We analysed data from four groups of participants – autistic participants with a formal diagnosis of autism, autistic participants without a formal diagnosis of autism, parents/carers, and members of autism-related organisations. With respect to gender, respondents were either male, female or other-identifying, the latter constituting participants who indicated ‘non-binary’ or ‘other’ in response to our question on gender. Participants who had indicated ‘other’ were also asked to describe their gender in free-form text; these text responses were analysed by a member of our research team and, if applicable, recategorised to one of the other predefined categories (male, female and non-binary), for e.g. when a participant selected ‘other’ and indicated that they were born female.

At the design phase of the survey, the decision was made to include autistic people without a formal diagnosis in order to be inclusive of the whole autistic community. However, it is also important to understand how this group’s experiences could differ from those of diagnosed autistic people. The decision to present data from those with and without a formal diagnosis was backed up by independent sample t-tests that showed significantly different results when comparing responses from these groups. For the question on which areas were important for change, a significantly greater proportion of participants with a formal diagnosis selected employment (M = 0.82, SD = 0.39), government funding for autism-specific services (M = 0.66, SD = 0.47), financial hardship (M = 0.55, SD = 0.50), support with gender, sexuality and relationship issues (M = 0.41, SD = 0.49), and post-diagnostic services (M = 0.71, SD = 0.45) compared to participants without a formal diagnosis (M = 0.76, SD = 0.43; M = 0.56, SD = 0.50; M = 0.48, SD = 0.50; M = 0.35, SD = 0.48; M = 0.59, SD = 0.49 respectively), all ts > 1.78, ps < 0.04. Formally diagnosed participants also prioritised the following areas significantly higher than participants with no formal diagnosis, with a lower rank number denoting higher importance: discrimination (M = 8.15, SD = 4.03 vs. M = 9.55, SD = 3.72) and post-diagnostic services (M = 9.71, SD = 5.39 vs. M = 11.35, SD = 5.80), all ts > -2.87, ps < 0.007, while they prioritised education significantly lower (M = 5.54, SD = 4.17 vs. M = 4.55, SD = 3.46), t(591) = 2.00, *p* = .02.

A member of the research team reviewed the free-form text responses given in the ‘Other’ category if/when selected as an area that was important for change. All ‘Other’ responses from non-English surveys were first translated into English using the same strategy as mentioned in above. The responses were then re-categorised as one or more of the other 18 areas in the list if applicable or remained as ‘Other’ if they did not fit with one of these areas. Another member of the research team evaluated the reclassification, and if there were disagreements, the reclassification was discussed until a consensus was reached.

For selection of priorities for change, results were presented as the percentage of participants selecting an area as a priority for change. The percentages were calculated across the whole sample as well as within each of the four participants groups and the five countries with the most participants in the sample – Germany, the UK, France, Spain and Poland. Overall ranking of the areas as priorities for change as well as the mean rank for each area, which is the average of the ranks assigned to an area by participants, were also derived within each of these samples. The overall rankings did not include ‘Other’ area and thus ranged from 1 to 18 as the option to rank it was only available to a small group of participants who had indicated it earlier as a priority for change. Mean ranks were calculated across all participants, regardless of whether they had ranked ‘Other’ area or not, so the maximum mean rank an area could be assigned to is 19.

For all autistic participants with or without formal autism diagnosis, gender-related differences in results were analysed via chi-squared independent tests. Only male and female participants were compared with each other due to the relatively small sample size of other-identifying participants (6.50%), which could restrict the validity of statistical tests if included. Chi-squared independent tests were also conducted to identify autism-related differences in results across the entire sample as well as within each group. Specifically, differences in results between autistic and non-autistic participants, between autistic participants with formal diagnosis and autistic participants with no formal diagnosis, between autistic and non-autistic parents/carers, and between autistic and non-autistic members of autism-related organisations were studied. If the expected count in a chi-squared test of independence was less than five in more than 20% of the cells, a Fisher’s exact test would be carried out instead. Gender-related and autism-related analyses were also performed when examining ranking of areas of priority for change, using Mann-Whitney U tests. Moreover, considering that the survey was open for 3.5 years (November 2020 – February 2024), we examined in the Supplementary Information if results were significantly affected by when the survey was completed, defining time as the number of years (treated as a continuous variable) between the survey launch date and the survey completion date. Binary logistic regressions were performed when analysing selection of priorities for change while ordinal logistic regressions were carried out for analyses on rankings. Sample size was controlled for in both sets of analysis, taking into account possible changes in the number of survey respondents over time. In addition, cross-country comparisons were conducted, analysing ranking results across the five most represented countries in the sample – Germany, the UK, France, Spain and Poland. We ran Kruskal-Wallis tests for these analyses, one within each of the four groups of participants, to determine if there was a significant difference in the ranking of each area across the five countries. Where results were significant, post-hoc Dunn’s tests with a Bonferroni correction for multiple tests were performed to identify between which countries the ranks significantly varied.

## Results

To keep the manuscript concise, only figures presenting the primary results of the study – namely, areas of priority for change and their ranking across the four participant groups – are included in the main text. Figures and tables illustrating other results cited in the main text as well as additional data are provided in the Supplementary Information.

### Demographics

Overall, 1,709 participants from the EU and the UK combined completed the *10 Points for Change* survey (see Supplementary Table 2; Supplementary Fig.s 3b-f), with most coming from Germany (31.77%; *n* = 542), the UK (24.27%; *n* = 414), France (11.20%; *n* = 191), Spain (8.73%; *n* = 149) and Poland (8.32%; *n* = 142). The average age of participants was 41.68 (range = 16–97; SD = 13.33; see Supplementary Fig. 3av) and 27.91% (*n* = 477) identified as male, 65.59% (*n* = 1,121) as female and 6.50% (*n* = 111) as other gender (see Supplementary Fig. 3ai). Participants in the other gender group comprised those who identified as either non-binary (72.97%; *n* = 81; 4.74% of total sample) or other gender type (27.03%; *n* = 30; 1.76% of total sample). Most participants (94.62%; *n* = 1,617) filled in the survey on their own but the rest (5.38%; *n* = 92) indicated that they completed it with support.

Just over half of participants were autistic (52.25%; *n* = 933) while 47.75% (*n* = 776) were non-autistic (see Supplementary Fig. 3aii). Out of the autistic respondents, most (84.03%; *n* = 784; 45.87% of total sample) said they were formally diagnosed with autism, while the remainder (*n* = 149; 15.97%; 8.72% of total sample) did not have a formal diagnosis. Participants without formal diagnosis comprised of those who reported themselves as self-diagnosed (53.69%; *n* = 80; 4.68% of total sample), awaiting assessment (25.50%; *n* = 38; 2.22% of total sample), or both (20.81%; *n* = 31; 1.81% of total sample).

Parents/carers constituted 43.83% (*n* = 749) of participants whereas one-fifth (20.60%; *n* = 352) of participants were members of autism-related organisations. Across all parents/carers, most (81.44%; *n* = 610; 35.69% of total sample) were non-autistic while 18.56% (*n* = 139; 8.13% of total sample) were autistic (see Supplementary Fig. 3aiii). The same pattern of distribution was seen for members of autism-related organisations, where the majority (73.01%; *n* = 257; 15.04% of total sample) were non-autistic while 26.99% (*n* = 95; 5.56% of total sample) were autistic (see Supplementary Fig. 3aiv).

More information on gender distribution across all autistic participants (with or without formal diagnosis), autistic participants with formal diagnosis, autistic participants with no formal diagnosis, parents/carers and members of autism-related organisations is in the Supplementary Information section (see Supplementary Fig. 4).

### Areas of priority for change

**Fig. 1:**
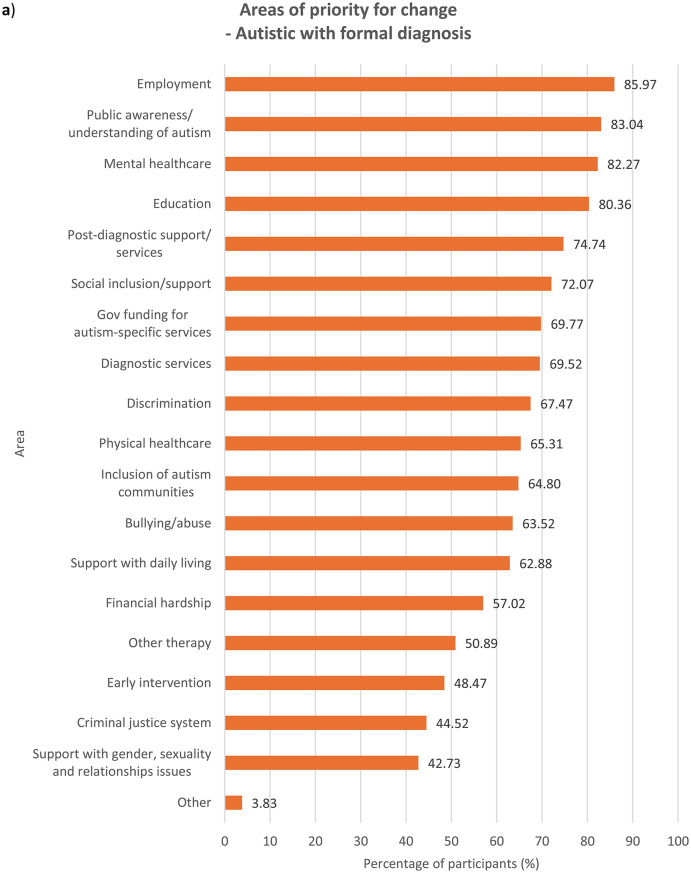

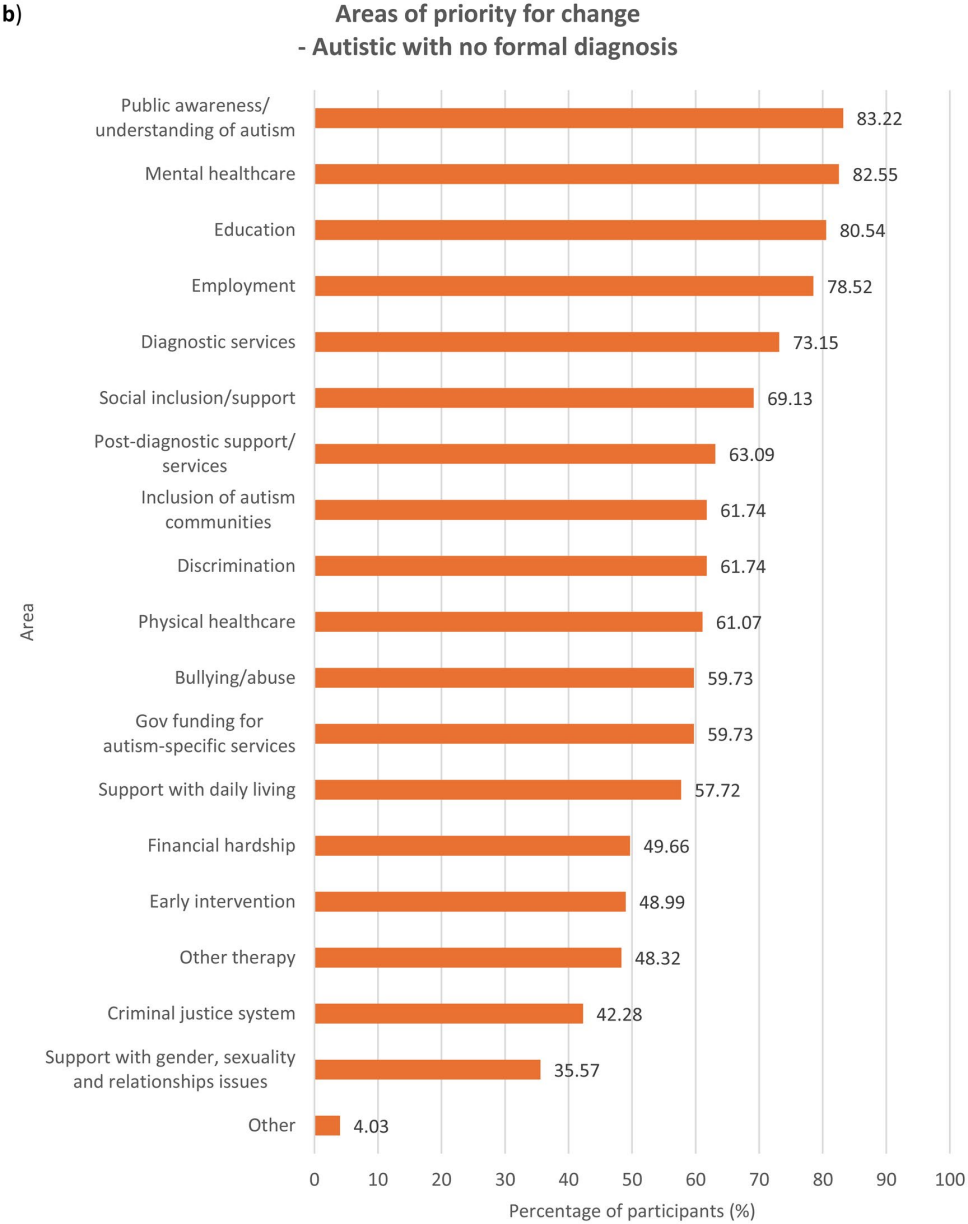

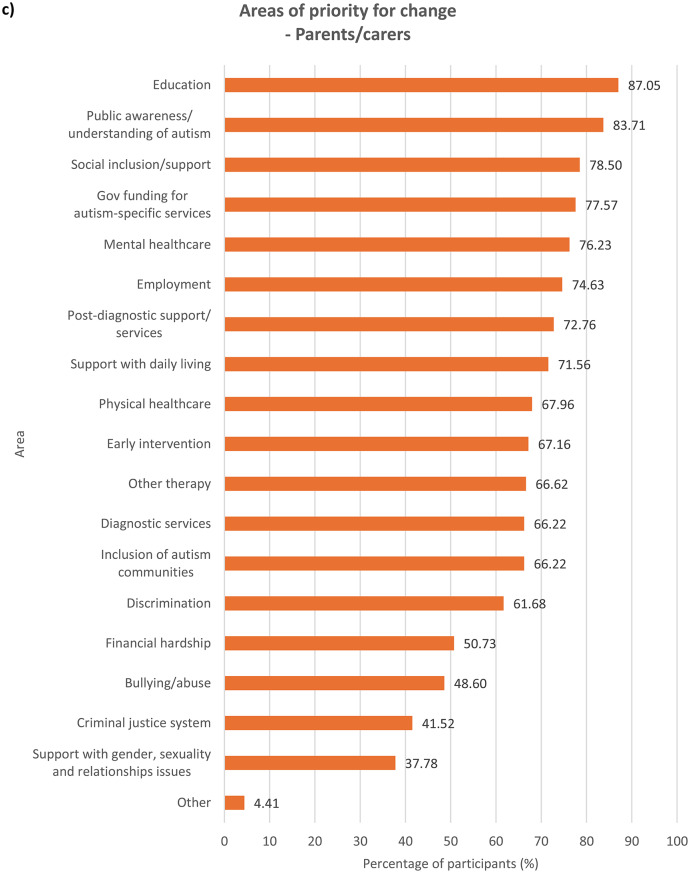

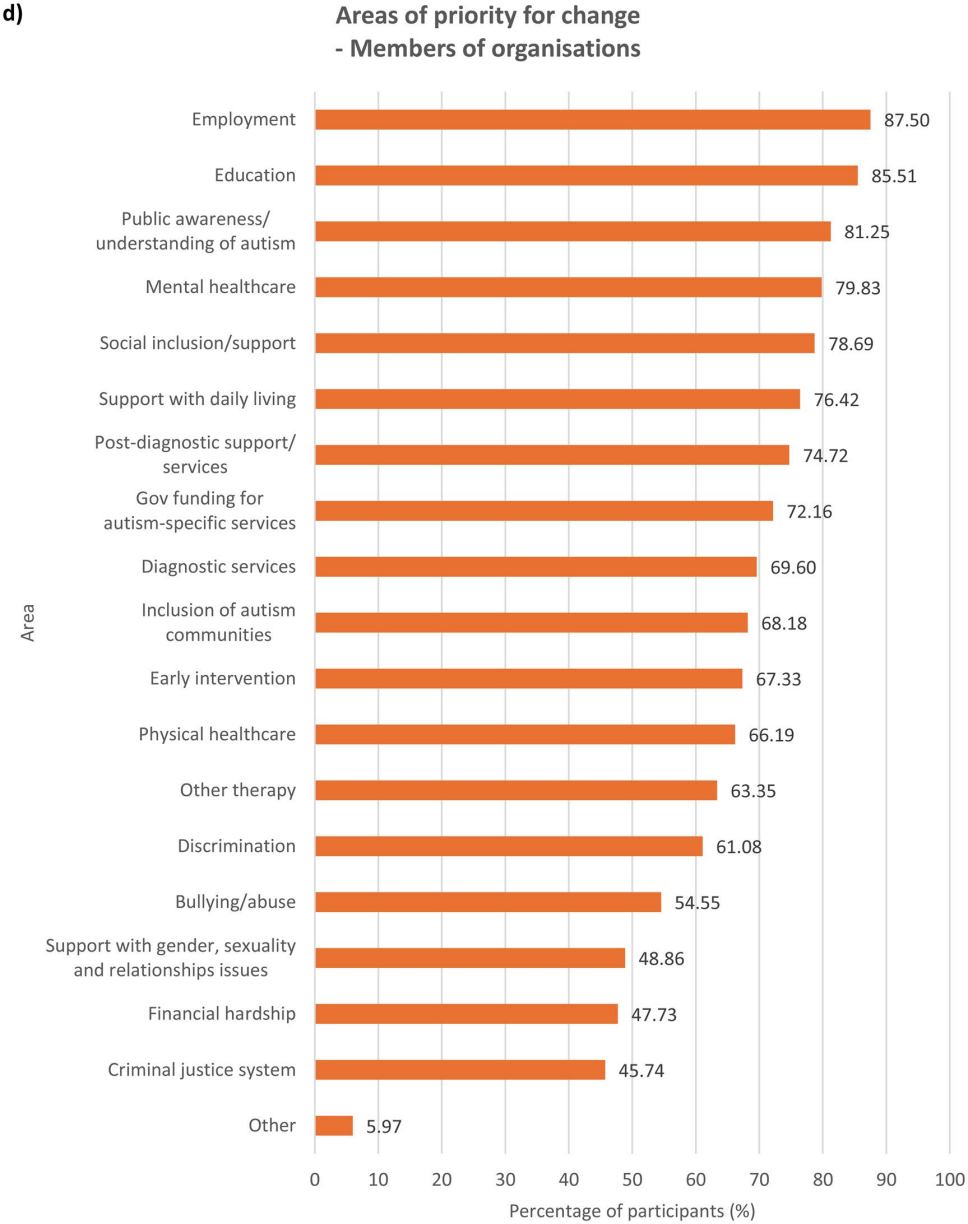
Areas that need to be changed for autistic people, according to **a**) autistic participants with a formal diagnosis of autism, *n* = 784, **b**) autistic participants with no formal diagnosis of autism (i.e. participants who were self-diagnosed and/or awaiting assessment), *n* = 149, **c**) parents/carers, *n* = 749, and **d**) members of autism-related organisations, *n* = 352. Multiple selections allowed

We asked participants to indicate, out of a list of 19 areas (see Supplementary Table 1), all areas they felt were important to change based on their experiences. Results were examined for each group of participants (see Fig. 1). In the Supplementary Information section, results across the entire sample (see Supplementary Fig. 11) as well as comparisons between autistic and non-autistic participants – across the whole sample (see Supplementary Fig. 5), among parents/carers (see Supplementary Fig. 7) and among members of autism-related organisations (see Supplementary Fig. 9) – were analysed.

When indicating ‘Other’ as an important area, the most reported theme was research, with some suggesting research into treatments and others commenting on the need for neurodiversity-affirming research. Other themes included adaptations to environments, difficulties related to COVID-19, access to information, compliance with EU disability policy, housing, policy/law changes, art resources, support with accessing benefits, removal of autism from the Diagnostic and Statistical Manual of Mental Health Disorders, Fifth Edition (DSM-5), respite care, elderly care, neurodiversity-affirming training and awareness, evaluating positive aspects of autism, access to sports, sibling support, recognition in girls, and holistic approaches.

### Ranking of areas of priority for change

At the end of the survey, participants were asked to rank the 19 areas (see Supplementary Table 1) in order of priority for change, where areas that required change the most were placed at the top while those which were less important for change were at the bottom. Ranks assigned to an area by participants were also averaged across participants within each group and across the entire sample, and the distribution of ranks for each area (see Supplementary Fig. 13) were calculated. For these analyses, rankings from participants who had also ranked ‘Other’ area were included.

**Fig. 2:**
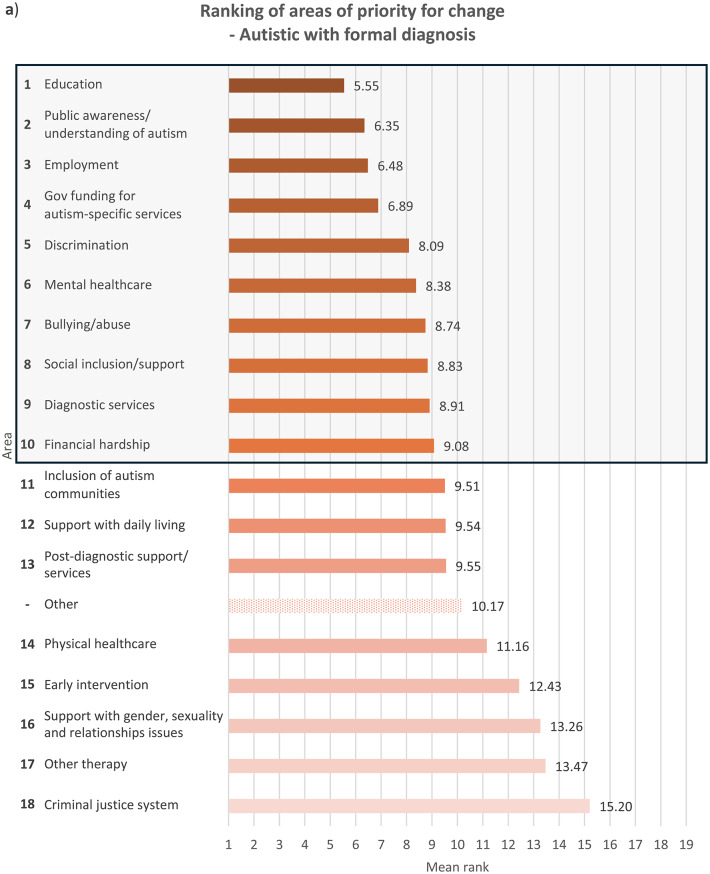

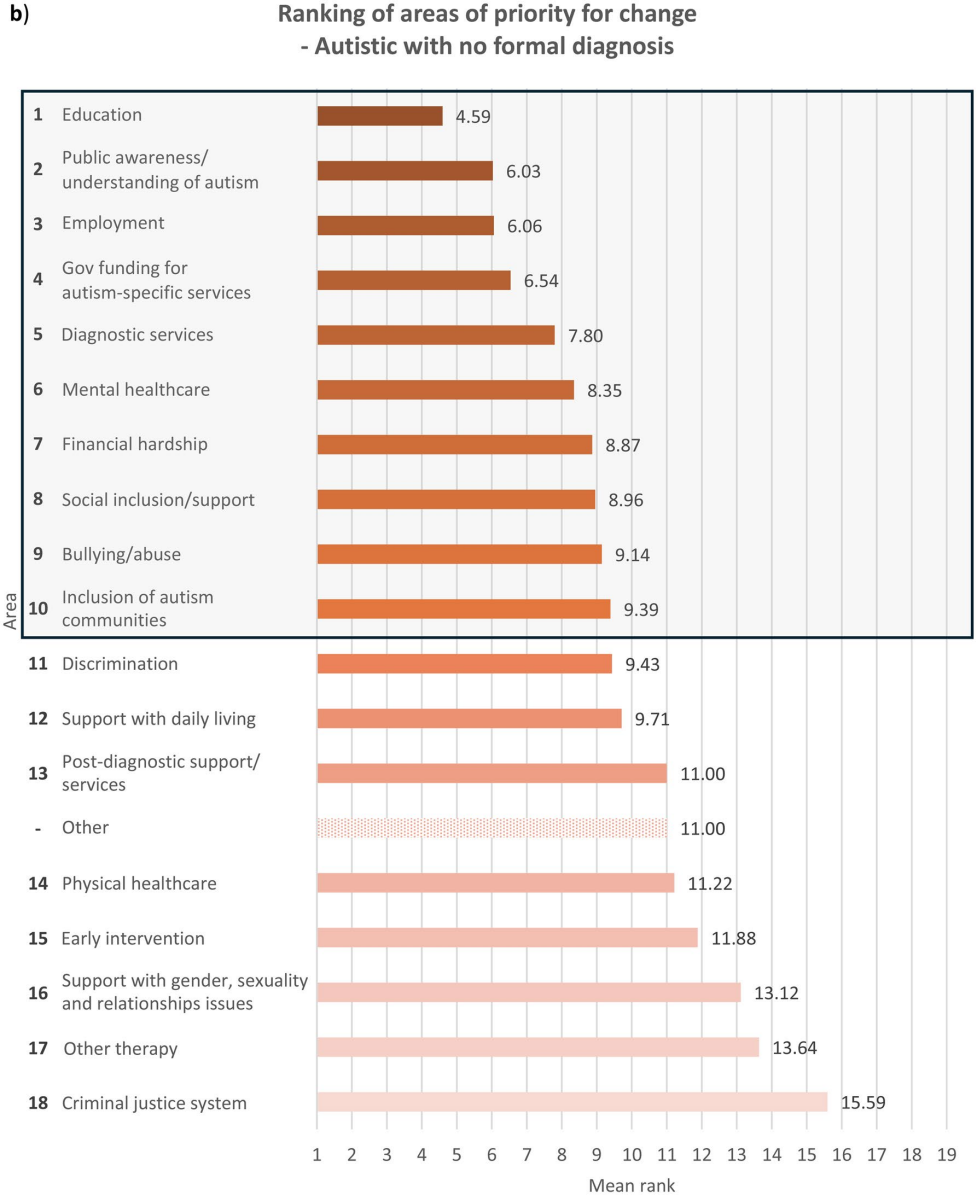

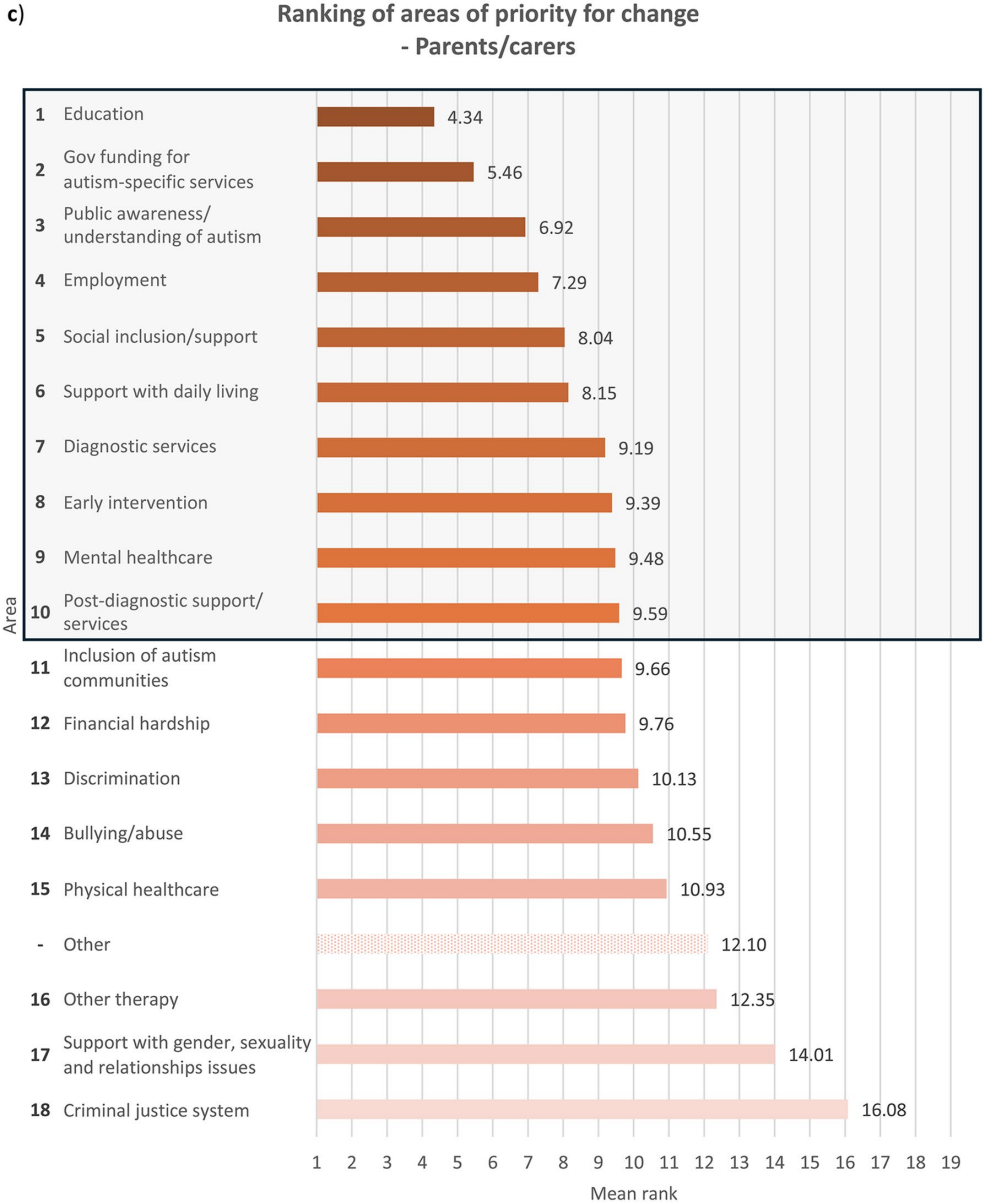

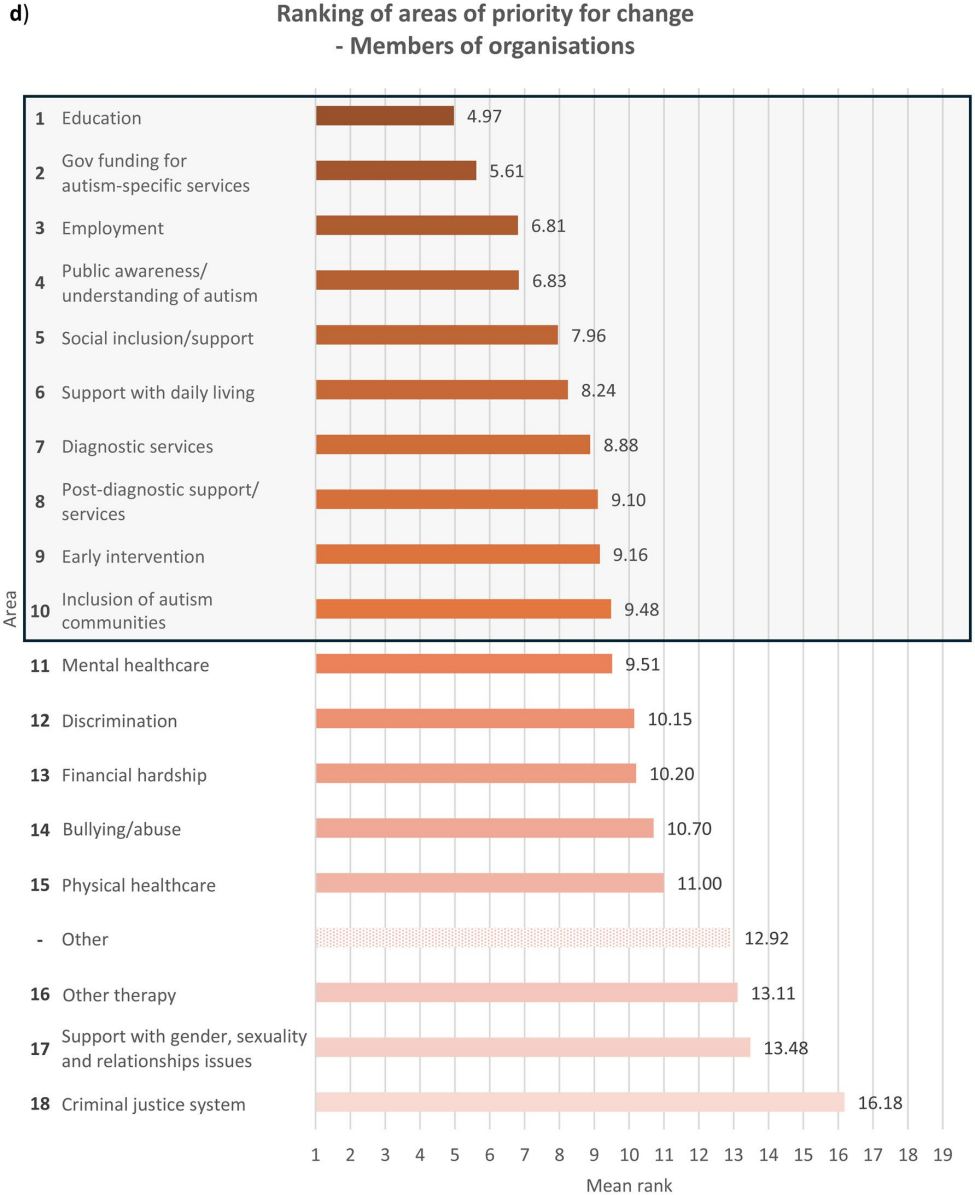
Overall ranking of areas that need to be changed for autistic people, with the top 10 highlighted in a box, in descending order of importance where 1 is most important and 18 least important (excluding ‘Other’ area), and mean rank of each area from 1 to 19 (including ‘Other’ area), indicated by **a**) autistic participants with a formal diagnosis of autism, *n* = 498; for ‘Other’ area, *n* = 23, **b**) autistic participants with no formal diagnosis of autism (i.e. participants who were self-diagnosed and/or awaiting assessment), *n* = 69; for ‘Other’ area, *n* = 3, **c**) parents/carers, *n* = 425; for ‘Other’ area, *n* = 20, and **d**) members of autism-related organisations, *n* = 205; for ‘Other’ area, *n* = 13. Overall ranking did not include ‘Other’ area due to the relatively small sample size of participants who ranked it, since only participants who had earlier in the survey indicated it as important for change were allowed to rank it, while those who had not were only asked to rank the other 18 areas

### Autism-related differences

Mann-Whitney U tests were conducted on each of the 19 areas to compare ranking trends across autistic participants (see Supplementary Fig. 6a) vs. non-autistic participants (see Supplementary Fig. 6b), autistic parents/carers (see Supplementary Fig. 8a) vs. non-autistic parents/carers (see Supplementary Fig. 8b), autistic members of autism-related organisations (see Supplementary Fig. 10a) vs. non-autistic members of autism-related organisations (see Supplementary Fig. 10b) and autistic participants with formal diagnosis (see Fig. 2a) vs. autistic participants with no formal diagnosis (see Fig. 2b). The full results are in Supplementary Table 3.

Autistic participants prioritised changes in the following areas significantly more than non-autistic participants: education, employment, financial hardship, public awareness/understanding of autism, discrimination, bullying/abuse, mental healthcare and the criminal justice system. In turn, non-autistic participants placed significantly more importance than autistic participants on changes in social inclusion/support, other therapy services, government funding for autism-specific services, support with daily living, and early intervention. No significant differences were reported in other areas between the two groups.

Within parents/carers, changes to bullying/abuse and diagnostic services were significantly ranked higher by autistic parents/carers than their non-autistic counterparts. Non-autistic parents/carers significantly ranked improvements in government funding for autism-specific services, social inclusion/support, support with daily living higher than autistic parents/carers. There was no significant difference between autistic and non-autistic parents/carers in their ranking of other areas.

Autistic members of autism-related organisations gave significantly higher priority to changes with regards to bullying/abuse, mental healthcare, and the criminal justice system compared to non-autistic members of autism-related organisations. On the other hand, improvement in education, government funding for autism-specific services, social inclusion/support, and early intervention was deemed significantly more urgent by non-autistic than autistic members of autism-related organisations. Both groups did not significantly differ when ranking other areas.

Among autistic participants, those with formal diagnosis prioritised changes in the area of discrimination significantly more than autistic participants without formal diagnosis. Contrariwise, autistic participants with no formal diagnosis put significantly greater importance on changes in diagnostic services than formally diagnosed autistic participants. Prioritisation of changes in other areas did not significantly differ between the two groups.

#### Gender-related differences

Significant differences in ranking trends between male and female participants within all autistic participants (with or without formal diagnosis) were also studied via Mann-Whitney U tests on all 19 areas of priority for change (see Supplementary Table 4).

Compared to female autistic participants, male autistic participants placed greater need for changes to employment, public awareness/understanding of autism, discrimination, bullying/abuse, social inclusion/support, support with gender, sexuality and relationship issues, and the criminal justice system. Female autistic participants prioritised improvement in government funding for autism-specific services, physical healthcare, mental healthcare, other therapy services, diagnostic services, and post-diagnostic support/services more than male autistic participants. No other significant differences in ranking results were observed.

#### Cross-country comparisons

Participants’ responses as to which areas were important for change (see Supplementary Fig.s 14–18) and how important they were for change (see Supplementary Fig.s 19–23, Supplementary Table 5, Supplementary Table 6) were compared across the five countries with the most participants in the survey – Germany, the UK, Spain, France and Poland (Supplementary Fig.s 3b-f).

The five countries in general showed common priorities for change within each participant group i.e. education, public awareness/understanding of autism, employment, and government funding for autism-specific services (see Supplementary Table 5). For formally diagnosed autistic participants as well as members of autism-related organisations, all countries except Poland have these four areas in their top five priorities for change. In Poland, autistic participants with formal diagnosis did not rank government funding for autism-specific services within their top five but ranked it 8th instead, whereas members of autism-related organisations did not indicate employment among their top five, though it was just outside of it (6th). This cross-country consistency was less prominent among autistic participants with no formal diagnosis, though education and public awareness/understanding of autism featured in all the countries’ top five. Top priorities for change according to parents/carers were also broadly consistent across all the five countries, where education, government funding for autism-specific services and public awareness/understanding of autism were reported among their top five.

The top five priorities for change were relatively consistent across all four groups of participants in Germany and the UK (see Supplementary Table 5), with education, public education/awareness of autism, government funding for autism-specific services, and employment present in all the groups’ top five. Priorities were also similar across the groups in France and Spain, though not as consistent, but greater cross-group variability was observed in Poland.

Kruskal-Wallis tests were also carried out to ascertain if the ranking of an area differed significantly across the five countries, and if results were significant, post-hoc Dunn’s tests with a Bonferroni adjustment for multiple tests were conducted to establish between which countries the significant difference was found (see Supplementary Table 6). For autistic people with formal diagnosis, significant country-related differences were observed in mean rank for employment (significantly more important in Spain vs. in Poland), government funding for autism services (significantly more important in the UK vs. in Germany), inclusion of autism communities in decision-making (significantly more important in Germany and the UK vs. in France), physical healthcare (significantly more important in France vs. in the UK), the criminal justice system (significantly more important in the UK vs. in Germany and France), diagnostic services (significantly more important in France vs. in the UK and Germany), post-diagnostic services (significantly more important in France vs. in Germany) and early intervention (significantly more important in France vs. in Germany). There was also an overall significant difference across the five countries for public awareness/understanding of autism, but post-hoc pairwise analyses revealed no significant pairwise comparisons upon Bonferroni correction. Cross-country differences in ranking for autistic participants with no formal diagnosis and members of autism-related organisations were not significant, but for parents/carers, the criminal justice system was significantly more important in the UK than in Germany.

## Discussion

This study set out to identify the top 10 priority areas for change for autistic people, according to various members of the autism community – autistic people with and without a formal diagnosis, parents and carers as well as members of autism-related organisations. Differences in the ranking of the priorities for change based on gender and autism diagnosis were also analysed. We furthermore assessed differences in results among countries with the largest representation in the survey – Germany, the UK, France, Spain and Poland. Common priorities for change were reported across most groups, with differences reflecting their specific needs and experiences.

### Priorities for change in Europe

With regards to ranking the top 10 areas that require change the most, all four groups of participants reported similar priorities (see Fig.s 2a-b). Education, public awareness/understanding of autism, government funding for autism-specific services, and employment figured in the top four priorities for all the groups, albeit in slightly different order. All the groups also ranked social inclusion and diagnostic services in the top 10. In comparison to parents/carers and members of autism-related organisations, autistic people with or without a formal diagnosis ranked bullying/abuse (7th for the formally diagnosed group and 10th for the undiagnosed group) and financial hardship (9th for the formally diagnosed group and 7th for the undiagnosed group) in the top 10 priorities for change, as opposed to bullying/abuse being ranked 14th by both parents/carers and members of autism-related organisations and financial hardship ranked 12th by parents/carers and 13th by members of autism-related organisations. On the other hand, parents/carers and members from autism-related organisations responded similarly to each other in terms of priority rankings. These groups prioritised support with daily living (both 6th), early intervention (8th for parents/carers and 9th for members of autism-related organisations) and post-diagnostic support (10th for parents/carers and 8th for member of organisations) in the top 10 areas for change, whereas these areas were less important for change for autistic participants with and without formal diagnosis, who both ranked the areas 12th, 15th and 13th respectively. This perhaps reflects the differing experiences of these groups, where a proportion of parents/carers and members of autism-related organisations were reporting on priorities relating to children or adults who could not report for themselves, and explains why early intervention, support with daily living and post-diagnostic services were seen as higher priorities.

Mental healthcare was a high priority for autistic participants with or without formal diagnosis (6th for both groups) as well as parents/carers (9th), but placed 11th among members of autism-related organisations. It is well-documented that mental health struggles are more prevalent among autistic people than among non-autistic people [14, 18], and autistic people encounter more challenges when attempting to access mental health support such as being declined due to systems not being set up to support people with other diagnoses such as autism [30]. Changes to accessibility of mental healthcare are therefore an utmost priority for autistic people and their families. However, the relatively low importance that members of autism-related organisations assigned to this area could stem from their interpretation that mental health issues faced by autistic people were attributed more to struggles in other areas such as education and social inclusion as opposed to primarily inefficiencies in mental healthcare. Other group-related differences in ranking were also observed (see **Discussion – Associations With Autism Diagnosis**, **Discussion** – **Associations With Gender**).

### Associations with autism diagnosis

When analysing ranking data (see **Results – Ranking of Areas of Priority for Change**), each group’s unique experiences contributed to significant differences in their prioritisation for change, though the data show less autism-related variation. Autistic participants ranked education, employment, financial hardship, public awareness/understanding of autism, discrimination, bullying/abuse, mental healthcare and the criminal justice system significantly more highly compared to non-autistic participants. On the contrary, non-autistic respondents viewed social inclusion/support, other therapy services, government funding for autism-specific services, support with daily living and early intervention as more urgent for change than did autistic participants. This distinction between autistic and non-autistic participants is perhaps spurred by the fact that all non-autistic participants in the survey were either parents/carers, members of autism-related organisations, or both, and so were more involved in areas they were responsible for, for example support with daily living. The parents and carers in our sample might also be reporting on their autistic family members with high support needs (and so were unable to represent themselves in the survey), and thus support with daily living and inclusion in the society were major concerns. The lack of availability and affordability of services in these areas profoundly affect many families across Europe, who also worry extensively about the future of their autistic loved ones ‘after us’, when they are no longer around to support them [34]. On the other hand, areas that were ranked significantly more highly by autistic participants than non-autistic participants were those that autistic people naturally know more about and have had personal experiences in, such as being bullied, compared to other non-autistic people including their own families and members of autism-related organisations.

Formally diagnosed autistic respondents assigned significantly higher importance to discrimination than did undiagnosed autistic participants, perhaps reflecting the prejudiced treatment the former received following the disclosure of their autism [35]. Autistic people with a formal diagnosis of autism are also more likely than those without one to have higher support needs or more observable autistic behaviours, which may have initially led to their referral for formal assessments, exposing them to discriminatory behaviour from others. Undiagnosed autistic participants, on the other hand, understandably ranked diagnostic services significantly more highly, which may be due to challenges faced by this group in accessing diagnostic services – a common problem among self-diagnosed or suspected cases of autism in children [36] and adults [37]. Autistic people with a formal diagnosis also ranked mental healthcare (6th) and bullying/abuse (7th) in the top 10 priorities for change, similar to autistic people without a formal diagnosis, though bullying/abuse was ranked 9th.

With regards to parents/carers and members of autism-related organisations, autistic participants were inclined to prioritise changes to areas which relate to their experiences as an autistic person significantly more than non-autistic participants from the same group. Autistic parents/carers, for example, placed significantly higher importance on changes in bullying/abuse than non-autistic parents/carers, as well as in diagnostic services, perhaps having gone through challenges with obtaining diagnostic services themselves. However, their non-autistic counterparts prioritised government funding for autism-specific services significantly more highly, possibly because non-autistic parents, carers and organisations are impacted by the costs of autism-specific services more than by other areas that they may have less direct experience in. Members of autism-related organisations also exhibited the same trend – autistic participants regarded discrimination as more important for change than did non-autistic participants, whereas government funding for autism-specific services were ranked significantly higher by non-autistic than autistic participants.

Even so, differences in how much two groups prioritise changes in an area do not necessarily suggest that it involves one group more than the other. Many areas, for example social inclusion/support and mental healthcare, concern a wide range of groups in various ways. One valuable implication from this is that enriching the experiences and wellbeing of autistic people impacts the whole community, autistic or non-autistic.

### Associations with gender

Gender effects were mixed and present in all groups for the ranking data (see **Results – Ranking of Areas of Priority for Change**). Male autistic participants with formal diagnosis ranked education, discrimination, bullying/abuse and social inclusion/support significantly more highly than female autistic participants with formal diagnosis; discrimination and bullying/abuse were also significantly stronger priorities for change among male autistic participants without formal diagnosis than their female counterparts. These results could primarily be attributed to the tendency for autistic boys and men to exhibit more visible and disruptive presentations than autistic girls and women [38], driving the assumption that male autistic students are in need of more assistance at school while female autistic students appear to be coping better [39]. Although autistic males ranked bullying higher as an area for change, bullying could be more covert among females than among males [40] (e.g. online and verbal insults vs. physical intimidation), explaining why more autistic girls reported being bullied [41]. On the other hand, female autistic participants with formal diagnosis gave significantly greater importance to changes in physical healthcare, mental healthcare, diagnostic services and post-diagnostic services than male autistic participants with formal diagnosis. Physical healthcare was also ranked significantly more highly by female autistic participants with no formal diagnosis than male participants in the same group. Compared to autistic males, autistic females have a greater risk of health problems [42] and undiagnosed or delayed diagnosis of autism [43] due to lack of clinical awareness of masking [44] and less obvious autistic traits [38, 45].

Although participants identifying as other gender were not included in gender-related analyses due to small sample size (6.50%), we acknowledge the greater barriers to services that gender-diverse and female autistic people have to overcome compared to male autistic people [46].

### Cross-country comparisons

There were many similarities in the ranking of priorities for change among the five countries with the greatest representation in our sample – Germany, the UK, France, Spain and Poland (see **Results – Cross-Country Comparisons**, Supplementary Table 5). In these countries, top priorities across participant groups were also generally similar. These findings highlight the common challenges faced by autistic communities across different countries in Europe and support the relevance and effectiveness of EU-level policies and multinational collaboration in addressing these challenges.

At the same time, we noted statistically significant country-specific differences across several areas (see Supplementary Table 6), primarily among autistic people with formal diagnosis and parents/carers. These results reveal that whilst there was overall consistency in the top five priorities for change across the countries, these differences perhaps reflect variation in services supporting autistic people across Europe, in particular with regards to government funding for autism services, diagnostic and post-diagnostic services, early intervention and healthcare provision. Differences in public awareness and understanding of autism across Europe may also impact participants’ perceptions of how important specific areas are for change. National policy responses people should therefore be tailored to the specific needs and contextual conditions of autistic populations within each country.

### Implications of ranking data

The ranking of the priorities for change (see **Results – Ranking of Areas of Priority for Change**) may carry different connotations and hence should be interpreted with caution. It is important to say that all of the areas examined in the survey are of priority to the autism community, which is why they were chosen based on our focus group discussions and existing literature during survey development. The highest-ranking areas for change are also those ranked highest by the most people and so are likely to be services or experiences that a high proportion of participants had and wanted to see changed. Education and employment, for instance, are critical periods of most autistic people’s lives and might therefore be considered urgent priorities for change by many more participants. Some of the priority areas that rank less highly may also be experienced by fewer people, but when experienced could be of high importance to these individuals. This can be inferred from data on what areas were selected by participants as requiring change (see **Results – Areas of Priority for Change**) as well as the distribution of ranks of the areas (see Supplementary Fig. 13). For example, the criminal justice system ranks last in all groups (see **Results – Ranking of Areas of Priority for Change**) and was the least frequently selected area by all of them (see **Results – Areas of Priority for Change**); however, other research indicates that some autistic people may be more vulnerable when involved with criminal justice professionals and that change is needed to improve awareness of autism among those working in law enforcement and the justice system [47].

Participants’ views on what areas should be changed and how important they are for change may also be influenced by how the areas relate to each other. Education and employment, for instance, are strongly interconnected – people’s experiences and opportunities in employment can substantially depend on their experiences in education [48, 49] – and are therefore perceived with comparable levels of importance. Similar links are also present between diagnostic and post-diagnostic services, as well as public awareness/understanding of autism, discrimination, bullying/abuse and social inclusion/support.

### Inclusivity of survey

Although there are concerns that autistic people with problems completing the survey for various reasons (e.g. young age, intellectual disability, physical difficulty) might not be included in the study, we believe our results offer some representation of the perspectives of autistic people of varying support needs and age. Data from parents and carers, completing the survey as themselves or for their autistic family members, should overlap with and correspond substantially to the views of their autistic relatives despite some differences [50, 51]. Because a large percentage of our participants comprised of parents and carers (43.83%; *n* = 749), the data from that group offer some opportunity to gather perspectives from the families of autistic people with high support needs. However, as this was not explicitly measured, we cannot definitively report on this. The number of participants indicating that they had filled in the survey with support (5.38%; *n* = 92; see **Methods and Materials – Participants**) might also be an underestimate, considering that the question could also be interpreted as asking whether the respondent themselves required assistance instead of the autistic person that the respondent was helping (see Supplementary Fig. 2). In addition, recruitment materials directly targeted carers alongside autistic people (see Supplementary Fig. 1) and did not specify any exclusionary criteria beyond age and residence (see **Methods and Materials** – **Participants; Methods and Materials** – **Survey Distribution**).

It is challenging for studies targeting large numbers of autistic people across a wide geographical area to capture data from participants with high support needs. However, the most effective method for such a reach, taking into account accessibility and convenience for both participants and researchers, remains conducting an online survey similar to *10 Points for Change* where parents and carers could complete the survey either on behalf of their autistic family members or as themselves. We acknowledge, nevertheless, that in our attempt to make the survey brief and manageable for participants, questions were not asked on the level of their support needs, on whether parents/carers were responding in the survey as themselves or for their autistic family members, and, if it was the latter, on whether their autistic family members were children or adults and what their level of support needs was. Future surveys should ensure these questions are included and participant reach is more inclusive (see **Discussion – Limitations**).

### Policy recommendations

As evidenced by the *10 Points for Change* survey, autistic people across Europe want to see changes in many areas of life. This may suggest that autistic people face challenges in these domains, encountering barriers to various services such as education, employment and healthcare services, and are denied reasonable accommodations [30]. It is important to note the intersectional dimension of the discrimination, as factors such as gender also influence the level of access to services [30]. Policy changes are therefore essential to addressing the needs of autistic people in Europe and should take the form of a coordinated effort across countries in the region.

Based on the findings of our survey, we established some key actions for change for autistic people in Europe. Our results have highlighted the need for enhanced access to services, aspects of society for autistic people, accommodations and reasonable adjustments; these could include but are not restricted to sensory adjustments to environments, flexibility in how appointments can be made and considering how services can be adapted to suit individual needs. Often, this requires a deeper understanding of the diversity of autistic people in terms of needs and priorities, and the recognition of the need for tailored solutions, which can primarily be achieved through systematic training and education in autism of people across sectors. Policies promoting training in all areas, ranging from schools and the workplace to healthcare and law enforcement, as well as funding for it, should be implemented to ensure accessibility of services, inclusion and the protection of the rights of autistic people. Support and inclusivity are important in enabling autistic people to meet their potential and contribute meaningfully to society. Conversely, there are personal quality of life and economic impacts of not addressing the needs of autistic people [52]. Where autistic people are not able to access reasonable adjustments, there need to be mechanisms in place for legal recourse and improved accountability. Future work should continue to engage with the autism community to better understand and design policy changes that meet the needs of autistic people.

The priorities highlighted in the *10 Points for Change* survey align with challenges identified by AE through various consultations across its membership, which includes over 90 associations from 40 countries representing autistic people and their families in Europe. AE has highlighted these challenges in an alternative report submitted to the Committee on the Rights of Persons with Disabilities (CRPD) in January 2025, regarding the status of the implementation of the UN Convention on the Rights of Persons with Disabilities for autistic people in the EU’s legislative and policy work [53]. Following the review, the CRPD has called on the EU to adopt new concrete actions to address the needs of autistic individuals in the second phase of the European Strategy for the Rights of Persons with Disabilities 2021–2030 [54]. The strategy is also in line with the European Parliament Resolution on Harmonising the Rights of Autistic Persons, adopted in 2023 [20]. The CRPD Recommendations include improving access to disability assessments for autistic people, combating poverty and social exclusion, promoting compulsory training of professionals on autism, and fostering quality support services to enable autistic people to live independently. Enhancing access to employment, including for people with intensive support needs, is also recommended.

In September 2024, the European Commission President announced the European Commissioners-designate as well as their portfolios and mission, including a commitment to work towards a ‘common approach’ to autism at the EU level as stated in the portfolio of the Commissioner for Health [55]. While this represents an important recognition, it is crucial to ensure that such an approach is not limited to a purely medical perspective on autism [56]. In line with international recommendations, such as the World Health Organisation (WHO) resolution entitled ‘Comprehensive and coordinated efforts for the management of autism spectrum disorders’ [57], the EU ‘common approach’ should promote at all levels of governance a holistic, rights-based, adequately funded and cross-sectoral policy response to address discrimination experienced by autistic people.

It is essential that any policies at the European, national, or regional level are designed and implemented in full cooperation with representatives of autistic people and their families. The *10 Points for Change* survey should serve as further guidance on the areas to be prioritised in future policies and funding. Having a clear picture of the situation experienced by autistic people – and its diversity across Europe but also local contexts – is fundamental to have a meaningful impact on the ground. It is therefore crucial to enhance efforts to systematically collect data about autistic people across Europe to monitor needs and progress.

To support this, we actively disseminated our findings to policymakers. On 23 April 2025, a high-level conference on autism took place at the European Parliament, organised by AE in partnership with our research team representing AIMS-2-TRIALS and endorsed by the Disability Intergroup of the European Parliament [58]. The conference titled ‘Towards a Common Approach for Autism in Europe’ aimed to foster a much-needed discussion among researchers, autism community representatives and Members of the European Parliament (MEPs) on developing a unified strategy across Europe for autistic people [53]. Results from the *10 Points for Change* survey as well as the ACCESS-EU survey, a study that examined autistic people’s access to services in Europe [30], were presented to highlight what changes are needed for autistic people according to autistic people, carers and members of autism-related organisations. Together with research from other AIMS-2-TRIALS teams on autistic people’s needs and challenges, essential input from representatives of the autistic community on actions that should be taken at the EU level, as well as speeches from MEPs supporting the creation of a binding strategy on autism, the event was a critical step in shedding light on the realities faced by autistic people across the region and the urgent need to protect their rights.

### Limitations

This research, however, is subject to several limitations related to its methodology. The areas shortlisted through focus group discussions for the survey might not have represented the experiences of all autistic people across the EU and the UK, but the data collected provided us with vital insights into autistic people’s lives, and sufficiently corresponded to most of the survey participants’ experiences (only 7.84% of participants selected ‘Other’ area; see Supplementary Fig. 11). While the survey, which was originally conceived in English, was translated into languages that were spoken by most residents in the EU and the UK – German, French, Italian, Spanish and Polish [29] – the choice to also provide Czech and Slovenian versions was based instead on the most common responses to a question in a previous survey with the same target participants (ACCESS-EU study) [30] as to what languages participants would like to see the survey in. In the face of budget constraints which restricted the number of languages the survey could be professionally translated to, language selection should have depended on the number of speakers in the region to maximise survey reach and represent the region more accurately. In this case, Romanian and Dutch translations would have been made available since Romania and the Netherlands were the 6th and 7th most populous countries respectively across the EU [59] and the UK [60]. Our rationale had been that more participants could be recruited by translating the survey into the most requested languages, and a high proportion of people in the Netherlands already speak English [29], so languages other than Dutch should be considered for translation. This might have also contributed to the underrepresentation of participants from Romania and the Netherlands in the survey (see Supplementary Table 2). Moreover, factors that are potentially crucial in influencing autistic people’s experiences such as socioeconomic status, level of support needs and ethnicity were not explored, hence they need further investigation in future research. Although age was asked in the survey, it was in relation to the respondents instead of the autistic people that parents, carers and members of autism-related organisations were referring to in their responses. Asking about the age of the autistic person in these cases would therefore be informative in future studies. It is furthermore not possible to discern, for participants who belonged to multiple groups, which group their responses were indicative of. An autistic parent, for example, might select diagnostic services as a priority for change, but this could be attributed to either their previous struggles as an autistic individual seeking a referral, or as a parent of a child waiting for assessment, or both. Participants’ overlapping identities should be taken into account in future iterations of the survey.

The analysis of non-English qualitative data should also be approached with caution. Non-English text responses in the quantitative sections of the survey (e.g. participants’ elaboration of what areas other than the ones listed in the survey were important to them for change) were translated into English using online translation tools such as Google Translate [31] and DeepL Translator [33], which might have been less reliable compared to native speakers. When applicable, ‘Other’ areas indicated by participants were also recategorised to the other predetermined areas based on their free-form text responses explaining what the areas were. Although data recategorisation was carried out by a member of the research team and checked by another, it is probable that a participant’s response was interpreted inaccurately or subjected to bias from the researchers.

Moreover, consideration should be given to the requirement for participants to rank all presented areas in order of importance for change, which might have assumed prematurely that they sought to observe changes in any of the areas and disregarded the possibility that no changes were desired at all. On a related note, asking participants which areas were important for change and then instructing them to rank all areas, regardless of whether the areas had been identified as priorities for change, made it more complex for data interpretation. It could be subjective as to which of the two types of data are more meaningful, though in this paper we decided to regard ranking results as a more precise indication of participants’ priorities for change. A more concise and accurate way of assessing the priorities is to ask participants to rank only areas that they thought needed to change (and, as discussed earlier, indicate if none of the areas applied).

Other issues pertinent to data representativeness should also be addressed. It is likely that autistic people with high support needs as well as autistic children are underrepresented in the study. Given that they were unable to complete the survey themselves, results from this study should be examined with the consideration that they might be skewed towards the experiences of more self-reliant autistic adults. Responses from parents and carers of autistic people, as well as members of autism-related organisations, might have also been biased towards their own views and might not include a representative cohort of those caring for autistic people with and without high support needs. However, while it is best to directly ask autistic people, regardless of their level of abilities, what their views are via more personal methods such as interviews and focus groups, surveys that also invite parents and carers to participate, such as the *10 Points for Change* survey, are the most efficient measure to assess the perspectives of large numbers of autistic people across multiple locations for the development of wide-reaching policies (see **Discussion** – **Inclusivity of Survey**) [61]. We had also circulated our survey through diverse channels ranging from social media to hospitals to increase the representativeness of the study. We acknowledge, nevertheless, that a bias towards autistic people with low support needs may still persist when using online surveys [62]. This is a challenge to autism research more broadly when collecting data from populations with higher support needs. Development of innovative methods is needed to be more inclusive of these populations in particular when collecting large-scale data [62]. In addition, it should be noted that the proportion of female participants in the sample (see Supplementary Fig. 3ai), even when examining only autistic groups (see Supplementary Fig. 4), was greater than those of male and other gender participants, although male preponderance in autism was commonly reported [63].

There may also be concerns with how representative the survey is of Europe. Even within the study sample, country distribution (see Supplementary Table 2) may not be fully representative of the population size distribution in the region comprising EU member states [59] and the UK [60]. Despite multiple attempts to disseminate the survey to underrepresented countries in the study by contacting local autism organisations for assistance with survey circulation, participant numbers remained low. In future surveys, it may be beneficial to reach out to non-autism-specific organisations as well such as medical centres and parent organisations as some autistic people might not be involved in autism-specific networks. The lack of representativeness of the sample might have also been caused by the number and type of languages that the survey was available in, as explained above. However, population size distribution may not always correspond with the proportions of autistic communities across the region due to unique issues that certain countries may face. In Slovenia, for example, where public awareness of developmental language disorders including autism is relatively low compared to Italy and Croatia, the reported prevalence of autistic people may be lower than in the latter countries [64].

We furthermore acknowledge concerns with data analysis involving small sample sizes. Group compositions for gender and autism diagnosis for some of the groups are limited by smaller samples sizes, for example the undiagnosed autistic group which was much smaller (8.72%) than the other groups. This may account for null findings for some of the group comparisons and these results should be interpreted with the caveat of a reduced sample size. For analyses on selection of priorities for change, Fisher’s exact tests were conducted instead of chi-squared tests of independence if the latter generated insufficient expected numbers of observations, but for ranking analyses, Mann-Whitney U tests would still work for small sample sizes, though the possibility of low power should be considered. Analyses on gender differences were also confined to male and female participants only, as the sample size of other-identifying participants (6.50%) was too small to be valid in statistical tests that compared them with much larger groups of male (27.91%) and female participants (65.59%). In future surveys, greater effort should be put into recruiting frequently underrepresented groups such as gender-diverse people, especially since gender diversity is more common among autistic than non-autistic people [65].

## Conclusion

Autistic people, parents and carers of autistic people, and members of autism-related organisations in Europe share similar views with respect to what areas need to change for autistic people. It is imperative that changes are made to education, public awareness and understanding of autism, government funding for autism-specific services, and employment to better address autistic people’s needs and improve their experiences. Differences in priorities for change were, however, observed among different members of the autistic community, reflecting their varied needs and experiences. Delaying the necessary changes needed to services and issues of importance to autistic people indicated in this survey risks both losing the numerous potential benefits that autistic people’s active participation in and contributions to the society can bring, and causing serious consequences to the mental health, overall wellbeing and quality of life of autistic people, as well as their families and members of their community.

## Supplementary Information

Below is the link to the electronic supplementary material.

Supplementary Material 1

## Data Availability

As further analyses of the data are underway and planned for later publication, the datasets generated and/or analysed in this study are not publicly available but are available from the corresponding author upon reasonable request.

## Abbreviations

ACCESS-EU: Autism Services Access – European Union
AE: Autism-Europe
AIMS-2-TRIALS: Autism Innovative Medicine Studies-2-Trials
A-Reps: Autism representatives
CARD: Cambridge Autism Research Database
CEPAMA: Comité para la Promoción y Apoyo de las Niñas y Mujeres Autistas (Committee for the Advancement and Support of Autistic Girls and Women)
CRPD: Committee on the Rights of Persons with Disabilities
DSM-5: Diagnostic and Statistical Manual of Mental Health Disorders, Fifth Edition
EU: European Union
MEP: Member of the European Parliament
UK: United Kingdom
WHO: World Health Organisation
