# Supplementary material for: Priorities for change for autistic people across Europe
Source: Mol Autism. 2026 Feb 19;17:12. doi: 10.1186/s13229-026-00706-3 (PMC12922334; doi:10.1186/s13229-026-00706-3)
Supplement: Supplementary file 1 — Supplementary Material 1 [file 13229_2026_706_MOESM1_ESM.docx]

# Supplementary Information

## **Table of Contents**

[Supplementary Information 1](#_Toc216702457)

[Survey Information 3](#_Toc216702458)

[Demographics 6](#_Toc216702459)

[Country Distribution 6](#_Toc216702460)

[Demographic Background 8](#_Toc216702461)

[Gender Distribution Within Group 13](#_Toc216702462)

[Group Differences in Ranking of Areas of Priority for Change 14](#_Toc216702463)

[Autism-Related Differences 14](#_Toc216702464)

[Gender-Related Differences 19](#_Toc216702465)

[Comparisons Between Autistic and Non-Autistic Participants 21](#_Toc216702466)

[All Participants 21](#_Toc216702467)

[Parents/Carers 26](#_Toc216702468)

[Members of Autism-Related Organisations 31](#_Toc216702469)

[Results Across All Participant Groups 36](#_Toc216702470)

[Areas of Priority for Change 36](#_Toc216702471)

[Ranking of Areas of Priority for Change 38](#_Toc216702472)

[Distribution of Ranking for Areas of Priority for Change 40](#_Toc216702473)

[Effect of Time Since Survey Launch on Results 43](#_Toc216702474)

[Areas of Priority for Change 43](#_Toc216702475)

[Ranking of Areas of Priority for Change 43](#_Toc216702476)

[Comparisons Across Top Five Countries in Sample 45](#_Toc216702477)

[Areas of Priority for Change 45](#_Toc216702478)

[Ranking of Areas of Priority for Change 70](#_Toc216702479)

## Survey Information


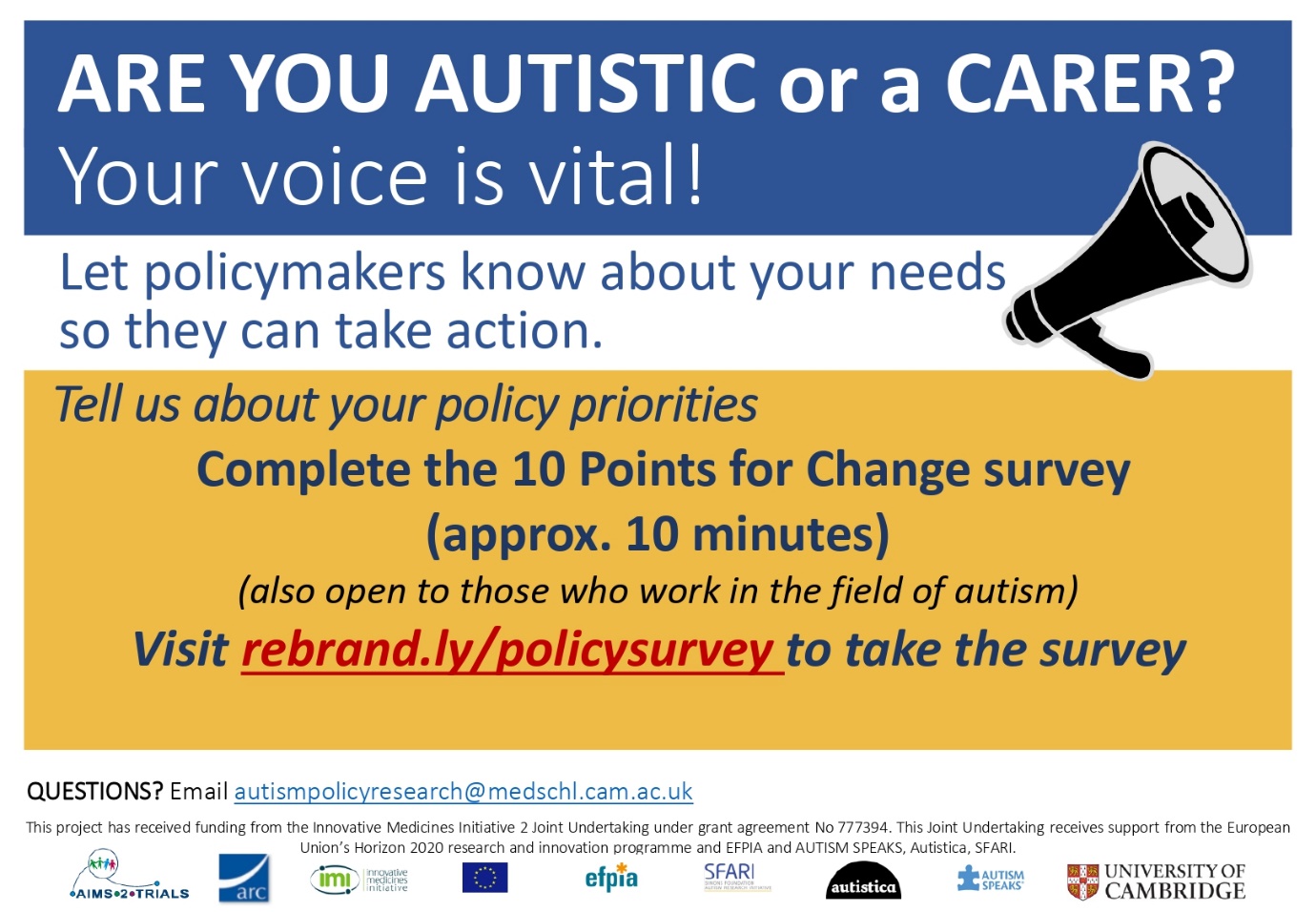


Supplementary Fig. 1. Recruitment poster for *10 Points for Change* survey in English. The poster was also translated by a translation agency into the seven other languages in which the survey was available i.e. Czech, French, German, Italian, Polish, Slovenian and Spanish.

Supplementary Table 1

*Areas of Priority for Change Examined in the Survey*

| Education |  |
| --- | --- |
| - E.g. mainstream vs. specialist, support/adaptations, choice, training |  |
| Employment |  |
| - E.g. finding and maintaining work, workplace support, equal opportunities |  |
| Government Funding for Autism-Related Services |  |
| - E.g. funding cuts, lack of resources, absence of services |  |
| Financial Hardship |  |
| - E.g. little/no disposable income, living in poverty, homelessness |  |
| Public Awareness and Understanding |  |
| - E.g. stigma, tokenism, stereotypes |  |
| Discrimination |  |
| - E.g. being treated negatively because of autism |  |
| Bullying/Abuse |  |
| - E.g. domestic abuse, victimisation, being manipulated |  |
| Social Inclusion/Support |  |
| - E.g. friendships, isolation, belonging, interacting with peers |  |
| Support With Daily Living |  |
| - E.g. support to live independently, managing a home, supported living |  |
| Support with Gender, Sexuality and Relationships Issues |  |
| - E.g. gender identity, sexual identity, forming and maintaining romantic relationships |  |
| Inclusion of Autism Communities (Autistic People/Carers/Organisations) in Decision-Making |  |
| - E.g. relating to policies, healthcare, housing, education |  |
| Physical Healthcare |  |
| - E.g. adaptations to services and appointments, accessibility, autism training for professionals |  |
| Mental Healthcare |  |
| - E.g. anxiety, depression, suicidality, therapy or support services, staff training |  |
| Other Therapy |  |
| - E.g. speech and language therapy, occupational therapy, behavioural therapies |  |
| The criminal justice system |  |
| - E.g. disclosure of diagnosis, reasonable adjustments, training for professionals |  |
| Diagnostic Services |  |
| - E.g. timely access, misdiagnosis, denial of a diagnosis, self-diagnosis |  |
| Post-Diagnostic Services |  |
| - E.g. adult support services, follow-up of diagnosis, providing information |  |
| Early Intervention |  |
| - E.g. any intervention for young children soon after autism diagnosis |  |
| Other |  |
| - Area(s) not presented in the list e.g. research | |
| *Note. These descriptors except for Other were shown to participants during the survey together with their respective areas.* | |


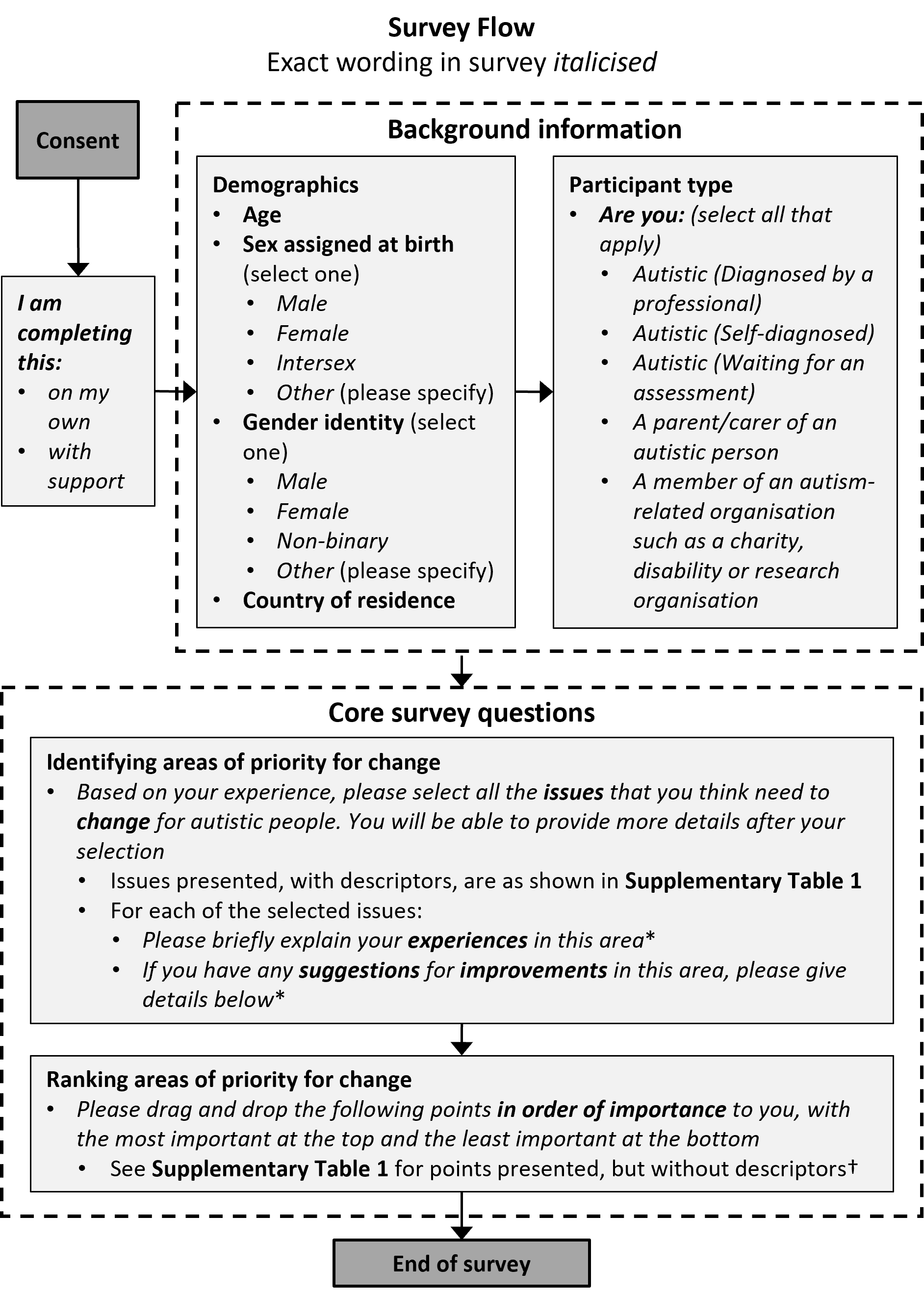


Supplementary Fig. 2. Flowchart of *10 Points for Change* survey. *Data were not analysed in this paper as they were qualitative and this paper only focused on quantitative data, which were the rest of the survey. †Only participants selecting ‘Other’ as an area of priority of change in the earlier section will be presented and asked to rank ‘Other’. Full survey is publicly available at https://osf.io/ucfhz/overview?view_only=c5d4b7d519f24e028d5b74f05289ab3f

## Demographics

### Country Distribution

Supplementary Table 2

*Distribution of Countries of Participants Within the Sample*

| **Country** | **Frequency** | **Percent (%)** |
| --- | --- | --- |
| Austria | 15 | 0.88 |
| Belgium | 20 | 1.17 |
| Croatia | 2 | 0.12 |
| Cyprus | 2 | 0.12 |
| Czech Republic | 21 | 1.23 |
| Denmark | 6 | 0.35 |
| Estonia | 0 | 0.00 |
| Finland | 6 | 0.35 |
| France | 191 | 11.20 |
| Germany | 542 | 31.77 |
| Greece | 3 | 0.18 |
| Hungary | 34 | 1.99 |
| Ireland | 20 | 1.17 |
| Italy | 42 | 2.46 |
| Latvia | 0 | 0.00 |
| Lithuania | 0 | 0.00 |
| Luxembourg | 4 | 0.23 |
| Malta | 7 | 0.41 |
| Netherlands | 33 | 1.93 |
| Poland | 142 | 8.32 |
| Portugal | 5 | 0.29 |
| Romania | 3 | 0.18 |
| Slovakia | 0 | 0.00 |
| Slovenia | 27 | 1.58 |
| Spain | 149 | 8.73 |
| Sweden | 21 | 1.23 |
| United Kingdom | 414 | 24.27 |
| **Total** | **1,709** | **100.00** |
| *Note. Only participants residing in European Union (EU) member states and the United Kingdom (UK) were included in the* 10 Points for Change *survey. While it may be important to consider the population size distribution across the region to ascertain the representativeness of the sample, autism prevalence may not be consistent with the breakdown of population sizes due to factors that can vary among countries such as public awareness of autism and the availability of local diagnostic services* | | |

### Demographic Background

**a**)

**EU**

**b**)

**Germany**

**c**)

**UK**

**d**)

**France**

**e**)

**Spain**

**f**)

**Poland**

Supplementary Fig. 3. a) Participants overall in the EU and UK. (i) Gender, n=1,709. (ii) Distribution of autistic (formally diagnosed or not formally diagnosed) and non-autistic participants, n=1,709. Participants with no formal diagnosis were defined as those who were self-diagnosed, awaiting assessment, or both. (iii) Distribution of autistic and non-autistic parents/carers, n=749. (v) Distribution of autistic and non-autistic members of autism-related organisations, n=352. (vi) Age distribution, n=1,709. b) Participants in Germany. (i) Gender, n=542. (ii) Distribution of autistic (formally diagnosed or not formally diagnosed) and non-autistic participants, n=542. Participants with no formal diagnosis were defined as those who were self-diagnosed, awaiting assessment, or both. (iii) Distribution of autistic and non-autistic parents/carers, n=172. (v) Distribution of autistic and non-autistic members of autism-related organisations, n=111. (vi) Age distribution, n=542. c) Participants in the UK. (i) Gender, n=414. (ii) Distribution of autistic (formally diagnosed or not formally diagnosed) and non-autistic participants, n=414. Participants with no formal diagnosis were defined as those who were self-diagnosed, awaiting assessment, or both. (iii) Distribution of autistic and non-autistic parents/carers, n=170. (v) Distribution of autistic and non-autistic members of autism-related organisations, n=52. (vi) Age distribution, n=414. d) Participants in France. (i) Gender, n=191. (ii) Distribution of autistic (formally diagnosed or not formally diagnosed) and non-autistic participants, n=191. Participants with no formal diagnosis were defined as those who were self-diagnosed, awaiting assessment, or both. (iii) Distribution of autistic and non-autistic parents/carers, n=88. (v) Distribution of autistic and non-autistic members of autism-related organisations, n=51. (vi) Age distribution, n=191. e) Participants in Spain. (i) Gender, n=149. (ii) Distribution of autistic (formally diagnosed or not formally diagnosed) and non-autistic participants, n=149. Participants with no formal diagnosis were defined as those who were self-diagnosed, awaiting assessment, or both. (iii) Distribution of autistic and non-autistic parents/carers, n=110. (v) Distribution of autistic and non-autistic members of autism-related organisations, n=45. (vi) Age distribution, n=149. f) Participants in Poland. (i) Gender, n=142. (ii) Distribution of autistic (formally diagnosed or not formally diagnosed) and non-autistic participants, n=142. Participants with no formal diagnosis were defined as those who were self-diagnosed, awaiting assessment, or both. (iii) Distribution of autistic and non-autistic parents/carers, n=86. (v) Distribution of autistic and non-autistic members of autism-related organisations, n=33. (vi) Age distribution, n=142.

### Gender Distribution Within Group

Supplementary Fig. 4. Percentage of male, female and other-identifying (non-binary and other genders) participants among all autistic participants (formally and not formally diagnosed; n=933), autistic participants with formal diagnosis (n=933), autistic participants with no formal diagnosis (n=149), non-autistic participants (n=776), parents/carers (n=749) and members of autism-related organisations (n=352). Autistic participants, parents/carers and members of autism-related organisations are overlapping groups i.e. a participant can belong to more than one of them. Autistic and non-autistic groups are mutually exclusive of each other, as are autistic with formal diagnosis and autistic with no formal diagnosis groups.

## Group Differences in Ranking of Areas of Priority for Change

### Autism-Related Differences

Supplementary Table 3

*Autism-Related Differences Among Participants in Ranking of Areas of Priority For Change*

| Group comparison | Participant sample | Results | Area | Mann-Whitney U test results |
| --- | --- | --- | --- | --- |
| Autistic vs. Non-autistic | **All participants** | **Autistic** participants prioritised changes in area significantly **more** vs. non-autistic participants | Education | z=-4.36, p<.001 |
|  |  |  | Employment | z=-2.95, p=.003 |
|  |  |  | Financial hardship | z=-2.46, p=.01 |
|  |  |  | Public awareness/ understanding of autism | z=-2.73, p=.006 |
|  |  |  | Discrimination | z=-8.25, p<.001 |
|  |  |  | Bullying/abuse | z=-7.75, p<.001 |
|  |  |  | Mental healthcare | z=-4.78, p<.001 |
|  |  |  | Criminal justice system | z=-5.31, p<.001 |
|  |  | **Non-autistic** participants prioritised changes in area significantly **more** vs. autistic participants | Social inclusion/ support | z=-4.23, p<.001 |
|  |  |  | Other therapy | z=-4.25, p<.001 |
|  |  |  | Government funding for autism-specific services | z=-7.01, p<.001 |
|  |  |  | Support with daily living | z=-5.80, p<.001 |
|  |  |  | Early intervention | z=-7.54, p<.001 |
|  |  | **No significant difference** between autistic and non-autistic participants in prioritising area for change | Support with gender, sexuality and relationship issues | z=-1.41, p=.16 |
|  |  |  | Inclusion of autistic communities in decision-making | z=-0.84, p=.40 |
|  |  |  | Physical healthcare | z=-0.81, p=.42 |
|  |  |  | Diagnostic services | z=-1.64, p=.10 |
|  |  |  | Post-diagnostic services | z=-0.16, p=.87 |
|  |  |  | Other | z=-0.42, p=.67 |
|  | **Parents/ carers** | **Autistic** parents/carers prioritised changes in area significantly **more** vs. non-autistic parents/carers | Bullying/abuse | z=-3.19, p=.001 |
|  |  |  | Diagnostic services | z=-2.24, p=.03 |
|  |  | **Non-autistic** parents/carers prioritised changes in area significantly **more** vs. autistic parents/carers | Government funding for autism-specific services | z=-2.67, p=.008 |
|  |  |  | Social inclusion/support | z=-2.41, p=.02 |
|  |  |  | Support with daily living | z=-2.44, p=.02 |
|  |  |  | Other therapy | z=-2.41, p=.02 |
|  |  | **No significant difference** between autistic and non-autistic parents/carers in prioritising area for change | Education | z=-1.81, p=.07 |
|  |  |  | Employment | z=-1.43, p=.15 |
|  |  |  | Financial hardship | z=-0.84, p=.40 |
|  |  |  | Public awareness/ understanding of autism | z=-1.01, p=.31 |
|  |  |  | Discrimination | z=-1.52, p=.13 |
|  |  |  | Support with gender, sexuality and relationship issues | z=-0.26, p=.80 |
|  |  |  | Inclusion of autistic communities in decision-making | z=-1.12, p=.27 |
|  |  |  | Physical healthcare | z=-1.68, p=.09 |
|  |  |  | Mental healthcare | z=-1.83, p=.07 |
|  |  |  | Criminal justice system | z=-0.97, p=.33 |
|  |  |  | Post-diagnostic services | z=-0.82, p=.41 |
|  |  |  | Early intervention | z=-1.27, p=.20 |
|  |  |  | Other | z=-0.04, p=.97 |
|  | **Members of autism-related organisations** | **Autistic** members of autism-related organisations prioritised changes in area significantly **more** vs. non-autistic members of autism-related organisations | Discrimination | z=-4.91, p<.001 |
|  |  |  | Bullying/abuse | z=-4.01, p<.001 |
|  |  |  | Mental healthcare | z=-2.57, p=.01 |
|  |  |  | Criminal justice system | z=-2.28, p=.02 |
|  |  | **Non-autistic** members of autism-related organisations prioritised changes in area significantly **more** vs. autistic members of autism-related organisations | Education | z=-2.89, p=.02 |
|  |  |  | Government funding for autism-specific services | z=-3.71, p<.001 |
|  |  |  | Social inclusion/ support | z=-2.08, p=.04 |
|  |  |  | Early intervention | z=-2.29, p=.02 |
|  |  | **No significant difference** between autistic and non-autistic members of autism-related organisations in prioritising area for change | Employment | z=-0.54, p=.59 |
|  |  |  | Financial hardship | z=-0.20, p=.85 |
|  |  |  | Public awareness/ understanding of autism | z=-0.26, p=.79 |
|  |  |  | Support with daily living | z=-1.90, p=.06 |
|  |  |  | Support with gender, sexuality and relationship issues | z=-1.71, p=.09 |
|  |  |  | Inclusion of autistic communities in decision-making | z=0.00, p=1.00 |
|  |  |  | Physical healthcare | z=-0.48, p=.63 |
|  |  |  | Other therapy | z=-0.34, p=.74 |
|  |  |  | Diagnostic services | z=-0.32, p=.75 |
|  |  |  | Post-diagnostic services | z=-0.37, p=.71 |
|  |  |  | Other | z=-0.83 p=.41 |
| Formally diagnosed vs. Not formally diagnosed | **Autistic participants** | **Formally diagnosed** autistic participants prioritised changes in area significantly **more** vs. non-formally diagnosed | Discrimination | z=-2.43, p=.02 |
|  |  | **Non-formally diagnosed** autistic participants prioritised changes in area significantly **more** vs. non-formally diagnosed autistic participants | Diagnostic services | z=-2.41, p=.02 |
|  |  | **No significant difference** between formally diagnosed and non-formally diagnosed autistic participants in prioritising area for change | Education | z=-0.60, p=.55 |
|  |  |  | Employment | z=-0.08, p=.93 |
|  |  |  | Government funding for autism-specific services | z=-0.50, p=.62 |
|  |  |  | Financial hardship | z=-0.23, p=.82 |
|  |  |  | Public awareness/ understanding of autism | z=-0.42, p=.68 |
|  |  |  | Bullying/abuse | z=-0.47, p=.64 |
|  |  |  | Social inclusion/ support | z=-0.22, p=.82 |
|  |  |  | Support with daily living | z=-0.54, p=.59 |
|  |  |  | Support with gender, sexuality and relationship issues | z=-0.43, p=.67 |
|  |  |  | Inclusion of autistic communities in decision-making | z=-0.54, p=.59 |
|  |  |  | Physical healthcare | z=-0.29, p=.77 |
|  |  |  | Mental healthcare | z=-0.32, p=.75 |
|  |  |  | Other therapy | z=-0.34, p=.73 |
|  |  |  | Criminal justice system | z=-0.61, p=.54 |
|  |  |  | Post-diagnostic services | z=-0.97, p=.33 |
|  |  |  | Early intervention | z=-0.05, p=.96 |
|  |  |  | Other | z=-0.44, p=.66 |

### Gender-Related Differences

Supplementary Table 4

*Gender-Related Differences Among Participants in Ranking of Areas of Priority For Change*

| Group comparison | Participant sample | Results | Area | Mann-Whitney U test results |
| --- | --- | --- | --- | --- |
| Male vs. Female | **Autistic participants** (with or without formal diagnosis) | **Male** autistic participants prioritised changes in area significantly **more** vs. female autistic participants | Employment | z=-2.49, p=.01 |
|  |  |  | Public awareness/ understanding of autism | z=-2.69, p=.004 |
|  |  |  | Discrimination | z=-4.69, p<.001 |
|  |  |  | Bullying/abuse | z=-4.20, p<.001 |
|  |  |  | Social inclusion/ support | z=-3.95, p<.001 |
|  |  |  | Support with gender, sexuality and relationship issues | z=-2.34, p=.01 |
|  |  |  | Criminal justice system | z=-2.06, p=.02 |
|  |  | **Female** autistic participants prioritised changes in area significantly **more** vs. male autistic participants | Government funding for autism-specific services | z=-2.42, p=.01 |
|  |  |  | Physical healthcare | z=-4.12, p<.001 |
|  |  |  | Mental healthcare | z=-4.52, p<.001 |
|  |  |  | Other therapy | z=-1.95, p=.03 |
|  |  |  | Diagnostic services | z=-3.33, p<.001 |
|  |  |  | Post-diagnostic support/services | z=-3.87, p<.001 |
|  |  | **No significant difference** between male and female autistic participants in prioritising area for change | Education | z=-0.81, p=.21 |
|  |  |  | Financial hardship | z=-0.21, p=.42 |
|  |  |  | Support with daily living | z=-0.57, p=.28 |
|  |  |  | Inclusion of autism communities in decision-making | z=-1.15, p=.13 |
|  |  |  | Early intervention | z=1.02, p=.85 |
|  |  |  | Other | z=0.08, p=.54 |

## Comparisons Between Autistic and Non-Autistic Participants

### All Participants

#### Areas of Priority for Change

Supplementary Fig. 5. Areas that need to be changed for autistic people, indicated by a) autistic participants, n=933, and b) non-autistic participants, n=776. Multiple selections allowed.

#### Ranking of Areas of Priority for Change

Supplementary Fig. 6. Overall ranking of areas that need to be changed for autistic people, in descending order of importance where 1 is most important and 18 least important (excluding ‘Other’ area), and mean rank of each area from 1 to 19 (including ‘Other’ area), indicated by a) autistic participants, n=84; for ‘Other’ area, n=7, and b) non-autistic participants, n=341; for ‘Other’ area, n=13. Overall ranking did not include ‘Other’ area due to the relatively small sample size of participants who ranked it, since only participants who had earlier in the survey indicated it as important for change were allowed to rank it, while those who had not were only asked to rank the other 18 areas.

### Parents/Carers

#### Areas of Priority for Change.

Supplementary Fig. 7. Areas that need to be changed for autistic people, indicated by a) autistic parents/carers, n=139, and b) non-autistic parents/carers, n=610. Multiple selections allowed.

#### Ranking of Areas of Priority for Change.

Supplementary Fig. 8. Overall ranking of areas that need to be changed for autistic people, in descending order of importance where 1 is most important and 18 least important (excluding ‘Other’ area), and mean rank of each area from 1 to 19 (including ‘Other’ area), indicated by a) autistic parents/carers, n=84; for ‘Other’ area, n=7, and b) non-autistic parents/carers, n=341; for ‘Other’ area, n=13. Overall ranking did not include ‘Other’ area due to the relatively small sample size of participants who ranked it, since only participants who had earlier in the survey indicated it as important for change were allowed to rank it, while those who had not were only asked to rank the other 18 areas.

### Members of Autism-Related Organisations

#### Areas of Priority for Change.

Supplementary Fig. 9. Areas that need to be changed for autistic people, according to a) autistic members of autism-related organisations, n=95, and b) non-autistic members of autism-related organisations, n=257. Multiple selections allowed.

#### Ranking of Areas of Priority for Change.

Supplementary Fig. 10. Overall ranking of areas that need to be changed for autistic people, in descending order of importance where 1 is most important and 18 least important (excluding ‘Other’ area), and mean rank of each area from 1 to 19 (including ‘Other’ area), indicated by a) autistic members of autism-related organisations, n=60; for ‘Other’ area, n=6, and b) non-autistic members of autism-related organisations, n=145; for ‘Other’ area, n=7. Overall ranking did not include ‘Other’ area due to the relatively small sample size of participants who ranked it, since only participants who had earlier in the survey indicated it as important for change were allowed to rank it, while those who had not were only asked to rank the other 18 areas.

## Results Across All Participant Groups

### Areas of Priority for Change

Supplementary Fig. 11. Areas that need to be changed for autistic people, indicated by participants across all groups (autistic participants with formal diagnosis, autistic participants with no formal diagnosis, parents/carers and members of autism-related organisations), n = 1,709. Multiple selections allowed.

### Ranking of Areas of Priority for Change

Supplementary Fig. 12. Overall ranking of areas that need to be changed for autistic people, in descending order of importance where 1 is most important and 18 least important (excluding ‘Other’ area), and mean rank of each area from 1 to 19 (including ‘Other’ area), indicated by participants across all groups (autistic participants with formal diagnosis, autistic participants with no formal diagnosis, parents/carers and members of autism-related organisations), n = 994; for ‘Other’ area, n = 40. Overall ranking did not include ‘Other’ area due to the relatively small sample size of participants who ranked it, since only participants who had earlier in the survey indicated it as important for change were allowed to rank it, while those who had not were only asked to rank the other 18 areas.

### Distribution of Ranking for Areas of Priority for Change

Rank

Rank

Rank

Supplementary Fig. 13. Percentage of participants (n=994) ranking, in descending order of importance where 1 is most important, areas that need to be changed for autistic people i.e. a) education, b) employment, c) government funding for autism-specific services, d) financial hardship, e) public awareness/understanding of autism, f) discrimination, g) bullying/abuse, h) social inclusion/support, i) support with daily living skills, j) support with gender, sexuality and relationship issues, k) inclusion of autism communities in decision-making, l) physical healthcare, m) mental healthcare, n) other therapy services, o) the criminal justice system, p) diagnostic services, q) post-diagnostic support/services, and r) early intervention, as well as s) ‘Other’ area, n=40. Range of rank is 1-18 for most participants, since most were not given the option to rank ‘Other’ area as they had not selected it as a priority for change earlier in the survey. Range of rank is 1-19 for participants who had selected ‘Other’ area as a priority for change and hence were able to rank it.

## Effect of Time Since Survey Launch on Results

Binary logistic regressions were carried out to determine if the selection of an area as a priority for change was significantly influenced by when the survey was completed. The effect on participants’ rankings of priorities of change was also studied, using ordinal logistic regressions. Significant results would suggest that priorities significantly changed over time, while the opposite would imply that priorities remained relatively the same throughout. In these analyses, the amount of time between when the survey was first available online and when it was completed by the participant was calculated as number of years, which is a continuous variable calculated down to the exact time of survey completion in seconds. Data were weighted based on the number of participants per month so that changes in sample size over time were controlled for.

### Areas of Priority for Change

Upon performing binary logistic regressions on the effect of time since survey launch on the likelihood of selecting an area, we found that in all groups, the model was not statistically significant for all areas. Odds ratios ranged from 0.93 to 1.85, all p ≥ .44 for autistic participants with formal diagnosis, 0.52 to 1.14, all p ≥ .62 for autistic participants with no formal diagnosis, 0.72 to 1.24, all p ≥ .61 for parents/carers, and 0.56 to 1.25, all p ≥ .56 for members of autism-related organisations.

### Ranking of Areas of Priority for Change

No significant associations were also yielded between time since survey launch and an area’s rank in all groups, with odds ratios ranging from 0.59 to 1.25, all p ≥ .39 for autistic participants with formal diagnosis, 0.40-1.82, all p ≥ .57 for autistic participants without formal diagnosis, 0.71-1.51, all p ≥ .63 for parents/carers, and 0.44-1.46, all p≥ .66 for members of autism-related organisations.

## Comparisons Across Top Five Countries in Sample

### Areas of Priority for Change

#### All Participants.

Supplementary Fig. 14. Areas that need to be changed for autistic people, indicated by participants across all groups (autistic participants with formal diagnosis, autistic participants with no formal diagnosis, parents/carers and members of autism-related organisations) in a) Germany, n=542, b) the UK, n=414, c) France, n=191, d) Spain, n=149 and e) Poland, n=142. Multiple selections allowed.

#### Autistic With Formal Diagnosis.

Supplementary Fig. 15. Areas that need to be changed for autistic people, indicated by autistic participants with formal diagnosis in a) Germany, n=283, b) the UK, n=247, c) France, n=97, d) Spain, n=20 and e) Poland, n=23. Multiple selections allowed.

#### Autistic With No Formal Diagnosis.

Supplementary Fig. 16. Areas that need to be changed for autistic people, indicated by autistic participants with no formal diagnosis in a) Germany, n=62, b) the UK, n=36, c) France, n=15, d) Spain, n=2 and e) Poland, n=13. Multiple selections allowed.

#### Parents/Carers.

Supplementary Fig. 17. Areas that need to be changed for autistic people, indicated by parents/carers in a) Germany, n=172, b) the UK, n=170, c) France, n=88, d) Spain, n=110 and e) Poland, n=86. Multiple selections allowed.

#### Members of Autism-Related Organisations.

Supplementary Fig. 18. Areas that need to be changed for autistic people, indicated by members of autism-related organisations in a) Germany, n=111, b) the UK, n=52, c) France, n=51, d) Spain, n=45 and e) Poland, n=33. Multiple selections allowed.

### Ranking of Areas of Priority for Change

#### Summary of Results.

Supplementary Table 5

*Top Five Priorities for Change Among Autistic Participants With Formal Diagnosis, Autistic Participants Without Formal Diagnosis, Parents/Carers and Members of Autism-Related Organisations, Comparing Results Across All Participants, Germany, the UK, Spain, France and Poland*

| AUTISTIC WITH FORMAL DIAGNOSIS | | | | |
| --- | --- | --- | --- | --- |
|  | **Overall** | **Germany** | **UK** |  |
| 1 | Education | Education | Government funding for autism-specific services |  |
| 2 | Public awareness/ understanding of autism | Public awareness/ understanding of autism | Education |  |
| 3 | Employment | Employment | Employment |  |
| 4 | Government funding for autism-specific services | Government funding for autism-specific services | Public awareness/ understanding of autism |  |
| 5 | Discrimination | Discrimination | Discrimination* |  |
|  | **Spain** | **France** | **Poland** |  |
| 1 | Employment | Education | Public awareness/ understanding of autism* |  |
| 2 | Government funding for autism-specific services | Diagnostic services | Education |  |
| 3 | Education | Employment | Diagnostic services |  |
| 4 | Public awareness/ understanding of autism | Government funding for autism-specific services | Support with daily living |  |
| 5 | Financial hardship | Public awareness/ understanding of autism | Employment |  |
| AUTISTIC WITH NO FORMAL DIAGNOSIS | | | | |
|  | **Overall** | **Germany** | **UK** |  |
| 1 | Education | Education | Education |  |
| 2 | Public awareness/ understanding of autism | Public awareness/ understanding of autism | Employment |  |
| 3 | Employment | Employment | Government funding for autism-specific services |  |
| 4 | Government funding for autism-specific services | Government funding for autism-specific services | Public awareness/ understanding of autism |  |
| 5 | Diagnostic services | Diagnostic services | Mental healthcare |  |
|  | **Spain** | **France** | **Poland** |  |
| 1 | Education | Education | Public awareness/ understanding of autism |  |
| 2 | Diagnostic services | Government funding for autism-specific services | Employment |  |
| 3 | Bullying/abuse | Diagnostic services | Government funding for autism-specific services |  |
| 4 | Discrimination | Financial hardship | Social inclusion/support |  |
| 5 | Public awareness/ understanding of autism | Public awareness/ understanding of autism | Inclusion of autism communities in decisions |  |
| PARENTS/CARERS | | | | |
|  | **Overall** | **Germany** | **UK** |  |
| 1 | Education | Education* | Education |  |
| 2 | Government funding for autism-specific services | Public awareness/ understanding of autism | Government funding for autism-specific services |  |
| 3 | Public awareness/ understanding of autism | Employment | Public awareness/ understanding of autism |  |
| 4 | Employment | Government funding for autism-specific services | Employment |  |
| 5 | Social inclusion/support | Social inclusion/support | Mental healthcare |  |
|  | **Spain** | **France** | **Poland** |  |
| 1 | Education | Education | Government funding for autism-specific services |  |
| 2 | Government funding for autism-specific services | Government funding for autism-specific services | Education |  |
| 3 | Social inclusion/support | Support with daily living | Public awareness/ understanding of autism |  |
| 4 | Public awareness/ understanding of autism | Public awareness/ understanding of autism | Support with daily living |  |
| 5 | Support with daily living | Post-diagnostic services | Employment |  |
| MEMBERS OF AUTISM-RELATED ORGANISATIONS | | | | |
|  | **Overall** | **Germany** | **UK** |  |
| 1 | Education | Education* | Government funding for autism-specific services |  |
| 2 | Government funding for autism-specific services | Public awareness/ understanding of autism | Education |  |
| 3 | Public awareness/ understanding of autism | Government funding for autism-specific services | Employment |  |
| 4 | Employment | Employment | Public awareness/ understanding of autism |  |
| 5 | Social inclusion/support | Social inclusion/support | Mental healthcare |  |
|  | **Spain** | **France** | **Poland** |  |
| 1 | Government funding for autism-specific services | Education | Government funding for autism-specific services |  |
| 2 | Education | Government funding for autism-specific services | Education |  |
| 3 | Support with daily living | Post-diagnostic services | Public awareness/ understanding of autism |  |
| 4 | Employment | Employment | Support with daily living |  |
| 5 | Public awareness/ understanding of autism | Diagnostic services | Social inclusion/support |  |

*Note. *’Other’ area was originally ranked here but not included in this table because ranking of ‘Other’ area was only available for participants who indicated it as important for change in an earlier survey question, hence only a subset of the sample ranked it while all participants ranked the other areas.*

#### **All Participants**.

Supplementary Fig. 19. Overall ranking of areas that need to be changed for autistic people, in descending order of importance where 1 is most important and 18 least important (excluding ‘Other’ area), and mean rank of each area from 1 to 19 (including ‘Other’ area), indicated by participants across all groups (autistic participants with formal diagnosis, autistic participants with no formal diagnosis, parents/carers and members of autism-related organisations) in a) Germany, n=331; for ‘Other’ area, n=8, b) the UK, n=244; for ‘Other’ area, n=12, c) France, n=117; for ‘Other’ area, n=8, d) Spain, n=86; for ‘Other’ area, n=2, and e) Poland, n=65; for ‘Other’ area, n=5. Overall ranking did not include ‘Other’ area due to the relatively small sample size of participants who ranked it, since only participants who had earlier in the survey indicated it as important for change were allowed to rank it, while those who had not were only asked to rank the other 18 areas.

#### Autistic With Formal Diagnosis.

Supplementary Fig. 20. Overall ranking of areas that need to be changed for autistic people, in descending order of importance where 1 is most important and 18 least important (excluding ‘Other’ area), and mean rank of each area from 1 to 19 (including ‘Other’ area), indicated by autistic participants with formal diagnosis in a) Germany, n=182; for ‘Other’ area, n=4, b) the UK, n=157; for ‘Other’ area, n=9, c) France, n=61; for ‘Other’ area, n=4, d) Spain, n=12; ‘Other’ area not included as n=0, and e) Poland, n=14; for ‘Other’ area, n=1. Overall ranking did not include ‘Other’ area due to the relatively small sample size of participants who ranked it, since only participants who had earlier in the survey indicated it as important for change were allowed to rank it, while those who had not were only asked to rank the other 18 areas.

#### Autistic With No Formal Diagnosis.

Supplementary Fig. 21. Overall ranking of areas that need to be changed for autistic people, in descending order of importance where 1 is most important and 18 least important (excluding ‘Other’ area), and mean rank of each area from 1 to 19 (including ‘Other’ area), indicated by autistic participants with no formal diagnosis in a) Germany, n=29; for ‘Other’ area, n=1, b) the UK, n=18; for ‘Other’ area, n=2, c) France, n=6; ‘Other’ area not included as n=0, d) Spain, n=1; ‘Other’ area not included as n=0, and e) Poland, n=5; ‘Other’ area not included as n=0. Overall ranking did not include ‘Other’ area due to the relatively small sample size of participants who ranked it, since only participants who had earlier in the survey indicated it as important for change were allowed to rank it, while those who had not were only asked to rank the other 18 areas.

#### Parents/Carers.

Supplementary Fig. 22. Overall ranking of areas that need to be changed for autistic people, in descending order of importance where 1 is most important and 18 least important (excluding ‘Other’ area), and mean rank of each area from 1 to 19 (including ‘Other’ area), indicated by parents/carers in a) Germany, n=109; for ‘Other’ area, n=2, b) the UK, n=93; for ‘Other’ area, n=6, c) France, n=57; for ‘Other’ area, n=5, d) Spain, n=66; for ‘Other’ area, n=2, and e) Poland, n=37; for ‘Other’ area, n=4. Overall ranking did not include ‘Other’ area due to the relatively small sample size of participants who ranked it, since only participants who had earlier in the survey indicated it as important for change were allowed to rank it, while those who had not were only asked to rank the other 18 areas.

#### Members of Autism-Related Organisations.

Supplementary Fig. 23. Overall ranking of areas that need to be changed for autistic people, in descending order of importance where 1 is most important and 18 least important (excluding ‘Other’ area), and mean rank of each area from 1 to 19 (including ‘Other’ area), indicated by members of autism-related organisations in a) Germany, n=64; for ‘Other’ area, n=2, b) the UK, n=35; for ‘Other’ area, n=2, c) France, n=35; for ‘Other’ area, n=5, d) Spain, n=25; for ‘Other’ area, n=2, and e) Poland, n=15; for ‘Other’ area, n=1. Overall ranking did not include ‘Other’ area due to the relatively small sample size of participants who ranked it, since only participants who had earlier in the survey indicated it as important for change were allowed to rank it, while those who had not were only asked to rank the other 18 areas.

#### Cross-Country Differences.

Supplementary Table 6

*Significant Differences Across Germany, the UK, France, Spain and Poland in Terms of Ranking of Areas of Priority for Change for Autistic People*

| Group | Area with significant cross-country differences | Mean rank | | | | | Results for overall differences | Results for pairwise differences | | |
| --- | --- | --- | --- | --- | --- | --- | --- | --- | --- | --- |
|  |  | **Germany** | **UK** | **France** | **Spain** | **Poland** | **Kruskal-Wallis** | **Country with higher priority for area** | **Country with lower priority for area** | **Post-hoc Dunn’s** |
| Autistic with formal diagnosis | Employment | 6.22 | 6.80 | 6.89 | 3.75 | 7.71 | χ^2^(4)=10.21, p=.04 | Spain | Poland | z=-2.82, p=.048 |
|  | Government funding for autism services | 7.68 | 6.08 | 7.18 | 4.92 | 8.57 | χ^2^(4)=17.56, p=.002 | UK | Germany | z=-3.50, p=.005 |
|  | Public awareness/ understanding of autism | 5.91 | 6.83 | 7.70 | 6.50 | 5.14 | χ^2^(4)=10.34, p=.04 | No significant differences | | |
|  | Inclusion of autism communities in decision-making | 9.06 | 9.20 | 11.75 | 9.33 | 9.07 | χ^2^(4)=15.28, p=.004 | Germany | France | z=-3.74, p=.002 |
|  |  |  |  |  |  |  |  | UK | France | z=-3.44, p=.006 |
|  | Physical healthcare | 10.77 | 11.68 | 9.77 | 13.17 | 11.36 | χ^2^(4)=13.47, p=.009 | France | UK | z=3.05, p=.02 |
|  | The criminal justice system | 15.62 | 14.32 | 16.13 | 13.92 | 16.07 | χ^2^(4)=25.71, p < .001 | UK | Germany | z=3.83, p=.001 |
|  |  |  |  |  |  |  |  | UK | France | z=-3.77, p=.002 |
|  | Diagnostic services | 9.48 | 9.16 | 6.25 | 9.33 | 7.43 | χ2(4)=18.61, p=.001 | France | UK | z=3.68, p=.002 |
|  |  |  |  |  |  |  |  | France | Germany | z=4.03, p=.001 |
|  | Post-diagnostic services | 10.30 | 9.29 | 8.00 | 8.83 | 10.30 | χ2(4)=10.06, p=.04 | France | Germany | z=3.02, p=.03 |
|  | Early intervention | 13.55 | 12.15 | 10.33 | 13.67 | 13.55 | χ2(4)=10.84, p=.03 | France | Germany | z=3.10, p=.02 |
| Autistic with no formal diagnosis | No significant differences | | | | | | | | | |
| Parents/ carers | The criminal justice system | 16.54 | 15.27 | 15.98 | 16.27 | 15.89 | χ2(4)=15.42, p=.004 | UK | Germany | z=3.09, p=.02 |
| Members of autism-related organisations | No significant differences | | | | | | | | | |

*Note. Post-hoc Dunn’s tests were conducted with Bonferroni adjustment for multiple tests. For non-significant comparisons, all ps ≥ .053 among autistic people with formal diagnosis, all ps ≥ .09 among autistic people with no formal diagnosis, all ps ≥ .054 among parents/carers, and all ps ≥ .08 among members of autism-related organisations. The mean rank of an area is the sum of ranks assigned to the area by participants in a group, divided by the number of participants. A lower rank assigned to an area means a higher priority for change, and vice versa.*
